# Left Ventricular Remodeling After Myocardial Infarction—Pathophysiology, Diagnostic Approach and Management During Cardiac Rehabilitation

**DOI:** 10.3390/ijms262210964

**Published:** 2025-11-12

**Authors:** Víctor Marcos-Garcés, Carlos Bertolín-Boronat, Héctor Merenciano-González, María Luz Martínez Mas, Josefa Inés Climent Alberola, Laura López-Bueno, Alfonso Payá Rubio, Nerea Pérez-Solé, César Ríos-Navarro, Elena de Dios, Jose Gavara, David Moratal, Jose F. Rodriguez-Palomares, Jose T. Ortiz-Pérez, Juan Sanchis, Vicente Bodi

**Affiliations:** 1 Department of Cardiology, Hospital Clinico Universitario de Valencia, 46010 Valencia, Spain; carlosbertolin7@gmail.com (C.B.-B.); hectormeren@gmail.com (H.M.-G.); mluzmmas@comv.es (M.L.M.M.); sanchis_juafor@gva.es (J.S.); vicente.bodi@uv.es (V.B.); 2 INCLIVA Health Research Institute, 46010 Valencia, Spain; neere_8@hotmail.com (N.P.-S.); cesar_rios1@hotmail.com (C.R.-N.); jose_4_6_90@hotmail.com (J.G.); 3 Network Biomedical Research Center for Cardiovascular Diseases (CIBER-CV), 28029 Madrid, Spain; elenaddll@gmail.com (E.d.D.); dmoratal@eln.upv.es (D.M.); jfrodriguezpalomares@gmail.com (J.F.R.-P.); 4 Department of Rehabilitation, Hospital Clinico Universitario de Valencia, 46010 Valencia, Spain; inescliment093@gmail.com (J.I.C.A.); laura.lopez@uv.es (L.L.-B.); paya_alf@gva.es (A.P.R.); 5 Centre for Biomaterials and Tissue Engineering, Universitat Politècnica de València, 46022 Valencia, Spain; 6 Department of Medicine, Universitat Autònoma de Barcelona, 08193 Barcelona, Spain; 7 Department of Cardiology, Hospital Universitari Vall d’Hebron, 08035 Barcelona, Spain; 8 Vall d’Hebron Institut de Recerca, 08035 Barcelona, Spain; 9 Institut d’Investigacions Biomèdiques August Pi i Sunyer (IDIBAPS), 08036 Barcelona, Spain; jtortiz@clinic.cat; 10 Cardiovascular Institute, Hospital Clínic, 08036 Barcelona, Spain; 11 Department of Medicine, Faculty of Medicine and Odontology, University of Valencia, 46010 Valencia, Spain

**Keywords:** left ventricular remodeling, cardiac rehabilitation, myocardial infarction, exercise training, echocardiography, cardiac magnetic resonance

## Abstract

Despite the improvement in prognosis in patients with acute myocardial infarction (AMI), a significant proportion of survivors still experience heart failure (HF)-related adverse outcomes. Adverse left ventricular remodeling (LVR), which refers to a progressive dilation of left ventricular (LV) end-diastolic and end-systolic volumes, usually accompanied by a deterioration in LV systolic function, occurs frequently and underlies most cases of HF development after AMI. In this review, we discuss the current definitions of post-AMI LVR, the most appropriate imaging modalities for its detection, and the pathophysiological mechanisms by which Cardiac Rehabilitation (CR) can improve LVR—including exercise interventions, cardiovascular risk factors control, and pharmacological therapy optimization. Finally, we provide up-to-date recommendations for the follow-up and management of LVR in post-AMI patients enrolled in CR and outline future prospects on this topic.

## 1. Introduction

Cardiovascular (CV) disease is the leading cause of mortality worldwide [1]. The World Health Organization estimates that nearly 20 million people died from CV disease in 2022, representing around one-third of all global deaths [2]. Among the entire spectrum of CV disease, ischemic heart disease accounts for most deaths and contributes substantially to morbidity. Acute myocardial infarction (AMI) represents one of its most severe manifestations, and substantial efforts have been made to improve its diagnosis, treatment and follow-up [2].

In recent decades, the prognosis of AMI patients has steadily improved [3]. Several factors have contributed to improved survival in the acute phase, such as the implementation of cardiopulmonary resuscitation, external defibrillation, coronary care units, and, more recently, thrombolysis and primary percutaneous coronary intervention (pPCI) [3]. Coronary angiography and revascularization are now standard procedures, tailored to clinical presentation and patient characteristics [4]. Patients meeting electrocardiographic or clinical criteria for ST-segment elevation or occlusion myocardial infarction (STEMI/OMI) require emergent coronary angiography (“AMI code”) [4].

The widespread adoption of pPCI in STEMI/OMI significantly improved short-term survival and reduced infarct size, leading to better long-term prognosis [3]. Pharmacological advances have also transformed care, establishing a comprehensive multidrug regimen as the current standard [5,6]. Post-AMI therapy should include antiplatelet agents to further prevent coronary thrombus formation; lipid-lowering therapy, including statins, to minimize or reverse atherosclerotic plaque progression; beta-blockers and renin–angiotensin–aldosterone system (RAAS) inhibitors to improve prognosis; and additional agents according to patient needs or the structural and functional consequences of AMI.

Given the importance of long-term CV risk factors control, current clinical practice guidelines emphasize the achievement of secondary prevention targets [7,8]. In this regard, Cardiac Rehabilitation (CR) is recommended as the most effective strategy to optimize risk factors control, improve quality of life, and enhance long-term prognosis [7,8]. Contrary to early 20th century recommendations of prolonged bed rest after AMI, one of the core components of CR is exercise training [9]. Beyond exercise, CR provides the ideal framework for pharmacological therapy optimization and dose titration, aimed at optimal secondary prevention achievement and prognostic improvement in patients with systolic dysfunction and/or heart failure (HF).

Improved acute-phase survival has, paradoxically, increased the prevalence of HF, which now represents a major contributor to adverse post-AMI outcomes [10,11]. Reduced left ventricular ejection fraction (LVEF) and adverse left ventricular remodeling (LVR) in the chronic phase are significant predictors of HF-related adverse events [12,13,14]. Identifying and treating patients with or at risk of LVR is therefore crucial to improving prognosis.

In this review, we discuss the contemporary diagnosis of LVR after AMI; the best imaging modalities and follow-up strategies in patients at risk of post-AMI LVR; and the pathophysiology and role of CR in modulating post-AMI LVR, from pharmacological, exercise-based, and CV risk factors perspectives. Our aim is to provide an up-to-date overview of the diagnosis and follow-up of LVR, while also addressing the implications of CR throughout this diagnostic and therapeutic process.

## 2. Search Strategy

For this narrative review, a comprehensive literature search was performed in PubMed/MEDLINE, Scopus, Google Scholar, and Web of Science databases for studies published up to October 2025. The following keywords and combinations were used: “myocardial infarction”, “ventricular remodeling”, “cardiac rehabilitation”, “exercise training”, “risk factor control”, and “pharmacological therapy”. Articles were limited to those published in English. We included original research articles, meta-analyses, and narrative or systematic reviews focusing on the definition, mechanisms, imaging assessment, and management of post-AMI LVR, as well as the role of CR interventions. Reference lists of relevant articles were also screened to identify additional publications. Given the narrative design of this review, no formal quality assessment or meta-analytic synthesis was performed.

## 3. Pathophysiology of Post-AMI Left Ventricular Remodeling

**Figure 1:**
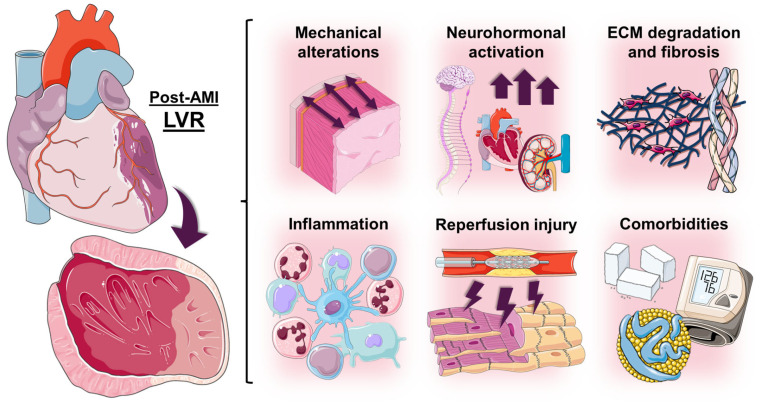
Pathophysiology of post-AMI LVR. Abbreviations: AMI = acute myocardial infarction. ECM = extracellular matrix. LVR = left ventricular remodeling.

Cardiac remodeling is a dynamic and complex process that arises after cardiac injury, usually due to obstruction of the epicardial coronary arteries, and involves structural and functional changes, particularly at the ventricular level [15,16] (Figure 1). As previously mentioned, these modifications are known to influence the patient’s clinical course, significantly increasing the risk of subsequent HF [17,18,19].

Acute coronary occlusion interrupts oxygen supply to the affected myocardium, leading to anaerobic metabolism, cell membrane destabilization, and cell death by apoptosis, autophagy, or necrosis [20]. Cell death begins in the subendocardial layers and progresses toward the subepicardial layers if the coronary blockage continues. Endothelial disruption also contributes to vascular permeability and tissue injury [21,22,23].

Massive cell death triggers an inflammatory response and cytokine release, which recruit leukocytes to the injured area. Metabolic and epigenetic mechanisms further regulate cell growth and apoptosis [24,25,26]. Certain cell lineages, such as macrophages, phagocytize dead cells and extracellular matrix debris as an initial step towards tissue repair, although excessive inflammation can lead to further expansion of the infarcted area [16,27,28,29,30].

In a later stage, fibroblasts generate a collagen matrix that forms fibrotic scar tissue [31,32]. Unlike some species capable of myocardial regeneration [33], mammals respond to AMI mainly by scar formation. Scar formation is generally regarded as positive since it prevents cardiac rupture and death, although excessive scarring increases wall stiffness, and the optimal balance required to achieve good mechanical and electrical scar properties is not yet defined [34,35]. Angiogenesis is another key response after AMI that contributes to tissue repair and has been targeted in cardiac regeneration studies [36,37].

At the same time, other biochemical processes are activated, such as the RAAS system, which stimulates proteolytic enzymes [25]. These biochemical cascades, together with mechanical mechanisms such as wall stress—with increased preload and afterload—progressively lead to wall thinning and ventricular dilatation and remodeling, thereby increasing the risk of developing HF.

### 3.1. Stages of Post-AMI LVR

LVR includes two phases: an early phase, occurring within the first days after AMI, and a late phase, mainly starting around one month later [38,39]. In the early phase, the infarcted zone stretches and thins due to the lack of contractile function, leading to an expansion of the infarcted area into neighboring regions. During this period, processes such as myocyte hypertrophy, apoptosis, and modifications to the extracellular matrix are also observed [38,40]. In the late phase, or chronic remodeling, the myocardium not directly affected by necrosis undergoes hypertrophy and dilation as an adaptive mechanism to increased wall stress. This response is highly variable and potentially reversible [40,41,42].

### 3.2. Factors Involved in the Pathophysiology of Post-AMI LVR

#### 3.2.1. Mechanical Alterations

Laplace’s law states ventricular wall tension is directly proportional to the pressure and ventricular radius, and inversely proportional to twice the wall thickness [18]. During early remodeling, stretching of the infarcted segment raises wall tension and promotes thinning. Additionally, the increase in left ventricular (LV) volume leads to volume and pressure overload in the non-infarcted areas. Initially compensated by the Frank–Starling mechanism, excessive dilation eventually leads to a self-perpetuating cycle of tension and expansion [38,42,43,44,45].

#### 3.2.2. Neurohormonal Activation

The sympathetic nervous system and the RAAS system induce vasoconstriction, fluid retention, hypertrophy, and fibrosis, perpetuating remodeling and worsening prognosis [46,47]. Endothelin is a vasoconstrictive peptide that promotes inflammation through different signaling pathways, contributing to adverse remodeling. It also stimulates cardiomyocyte hypertrophy [48,49]. Natriuretic peptides exert a protective effect through natriuresis, vasodilation, and inhibition of the RAAS and sympathetic nervous system [49]. In addition, they may regulate hypertrophy and have a well-known prognostic role in HF [50]. Indeed, reducing the harmful effects of neurohormonal activation is one of the main goals of current pharmacological therapy for HF [51,52].

#### 3.2.3. Extracellular Matrix Degradation and Fibrosis

After AMI, matrix metalloproteinases (MMPs) initially degrade the extracellular matrix, followed by collagen deposition from myofibroblasts [16,27,28,29,30]. Fibrosis can be reversible (interstitial) or irreversible (replacement), and its excess may compromise oxygen exchange [53]. Extensive scarring is detrimental, but several additional factors such as scar transmurality, collagen bundle orientation, and wall mechanical properties can also impact LVR beyond the extent of scarring [34,54].

#### 3.2.4. Inflammation

AMI-induced cell death releases intracellular components that activate the immune system, triggering a significant inflammatory response. This initial response is beneficial and necessary for myocardial repair [25]. However, a chronic proinflammatory state can drive maladaptive remodeling. Several cytokines, such as interleukin-1β, interleukin-6, and tumor necrosis factor (TNF)-α, amplify tissue damage, thereby promoting adverse LVR [18,55].

#### 3.2.5. Reperfusion Injury

Emergent coronary reperfusion is essential to reduce infarct size and improve long-term prognosis and LV systolic function. However, in some cases coronary reperfusion can worsen acute myocardial damage in a phenomenon known as ischemia–reperfusion injury [56]. One of the mechanisms involved is the production of reactive oxygen species (ROS) which, in addition to enhancing inflammation, can directly damage cell membrane lipids and deoxyribonucleic acid, leading to cell death [57,58].

#### 3.2.6. Comorbidities

Post-infarction cardiac remodeling is aggravated by various comorbidities and contributing factors. Chronic kidney disease can promote adverse LVR through increased hemodynamic load, dysregulation of the RAAS system, uremic toxins, anemia, and pro-inflammatory states [59,60]. Hypercholesterolemia, hypertension, type 2 diabetes, obesity, and smoking can also impact post-AMI LVR. Please refer to Section 5 for further information.

## 4. Definition and Diagnosis of Post-AMI Left Ventricular Remodeling

### 4.1. Definition of Post-AMI LVR

**Figure 2:**
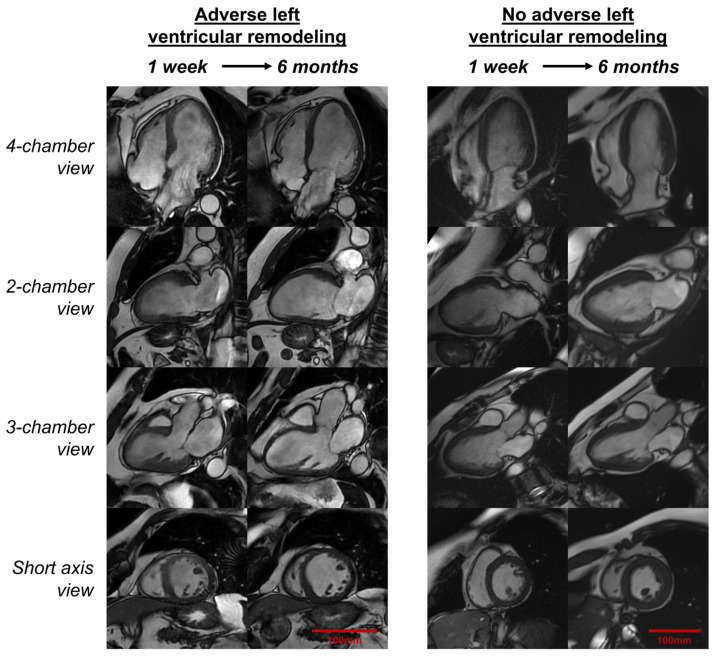
Representative examples of LVR post-AMI. On the left side, a case of a 70-year-old male who presented with anterior STEMI. Early (1-week) CMR showed extensive infarction in 8 segments of the left anterior descending artery territory, normal LVEDV (94 mL/m^2^), dilated LVESV (75 mL/m^2^), and severely reduced LVEF (21%). CMR in chronic phase (6 months) depicted signs of adverse LVR (LVEDV 130 mL/m^2^, +38%; LVESV 91 mL/m^2^, +21%) and reduced LVEF (31%). On the right side, a case of a 67-year-old woman who presented with inferior STEMI. Early (1-week) showed transmural infarction in 3 segments and subendocardial infarction in 2 segments of the right coronary artery territory, normal LVEDV (70 mL/m^2^) and LVESV (44 mL/m^2^), and moderately reduced LVEF (38%). CMR in chronic phase (6 months) depicted no signs of adverse LVR (LVEDV 64 mL/m^2^, LVESV 31 mL/m^2^) and improvement of LVEF (51%). Abbreviations: AMI = acute myocardial infarction. CMR = cardiac magnetic resonance. LVEDV = left ventricular end-diastolic volume. LVEF = left ventricular ejection fraction. LVESV = left ventricular end-systolic volume. LVR = left ventricular remodeling.

Although post-AMI LVR is generally defined as a change in LV volumes over time, there is still no universally accepted definition [61]. The lack of clear and consistent criteria makes it difficult to precisely establish what is meant by LVR, when and how follow-up should be performed, which factors are associated with it, how often it occurs, and what its prognostic value is in patients with AMI. The absence of consensus has contributed to marked variability in study results and the abundance of research with diverse designs and definitions (Figure 2, Table 1).

Different cardiac imaging methods can be used for the diagnosis of LVR: echocardiography, cardiac magnetic resonance (CMR), and nuclear techniques. Echocardiography is widely available and serves as the first-line method, but it has limitations in spatial resolution and reproducibility [18]. CMR imaging, while less accessible and more complex, provides more accurate and reproducible measurements and is considered the gold standard for assessing LV volumes and systolic function [62,63]. For this reason, in recent years CMR has become the reference technique for quantifying LVR [64,65].

Studies published to date use different parameters and thresholds to define LVR [17,61,66,67,68,69]. The most common are the percentage changes in left ventricular end-diastolic volume (LVEDV) and left ventricular end-systolic volume (LVESV). However, the thresholds initially used were derived from echocardiographic studies conducted mostly before the era of pPCI and were not initially strongly linked to major clinical events [70,71]. Furthermore, the optimal timing for post-infarction imaging follow-up to assess LVR is still debated. Studies using serial CMR have shown that volumes can remain relatively stable during the first month after STEMI, with a significant increase in volumes occurring between the first and third month [72]. Published studies are highly heterogeneous in this regard. Some analyze the first 3–4 months after infarction, others focus on around 6 months, while some include longer follow-up periods [61].

**Table 1:** Echocardiographic and CMR definitions of post-AMI LVR.

Study	Year	*n*	Imaging Modality	LVR Criteria			Follow-Up Timing	Endpoint
				∆LVEDV	∆LVESV	∆LVEF		
Bolognese et al. [17]	2002	284	TTE	>20%	-	-	6 months	Cardiac death and aHF
Mannaerts et al. [73]	2004	33	TTE	>20%	-	-	6 or 12 months	Prediction of LVR
van der Bijl et al. [67]	2020	1995	TTE	>20%	-	-	3, 6, or 12 months	aHF
Silveira et al. [74]	2021	50	TTE	≥15%	(and/or) ≥15%	-	6 months	Prediction of LVR
Logeart et al. [75]	2024	410	TTE	>20%	-	-	6 months	All-cause death or aHF
Bulluck et al. [76]	2017	40	CMR	≥12%	(and) ≥12%	-	5 months	Prediction of LVEF < 50%
Rodriguez-Palomares et al. [77]	2019	374	CMR	>15%	-	(and) ↓ > 3%	6 months	CV death, aHF or VA
Alonso Tello et al. [78]	2025	1067	CMR	>15%	-	(and) ↓ > 3%	6 months	CV death, aHF or VA
Reindl et al. [15]	2019	224	CMR	≥10%	-	-	4 months	All-cause death, AMI, stroke, or HF
Bulluck et al. [79]	2020	285	CMR	≥12%	(and) ≥12%	-	6 months	All-cause death or aHF
Shetelig et al. [80]	2018	240	CMR	≥10 mL/m^2^	-	-	4 months	Association with interleukin-8 levels
Garg et al. [81]	2017	50	CMR	-	>15%	-	3 months	Worsening of systolic function
Shetye et al. [82]	2017	65	CMR	≥20%	(and/or) ≥15%	-	4 months	Prediction of LVR
Sugano et al. [83]	2017	71	CMR	>5%	-	-	6 months	Prediction of LVR
Fabregat-Andrés et al. [84]	2015	31	CMR	>10%	-	-	6 months	Association with PGC-1α levels
Huttin et al. [85]	2017	121	CMR	>17.3 mL	-	(or) ↓ > 8.3%	6 months	Association with vascular function
Eitel et al. [86]	2011	154	CMR	-	Any ↑	Any ↓	6 months	Usefulness of intracoronary abciximab application

“↓” indicates reduction. Abbreviations: aHF = admission for heart failure. AMI = acute myocardial infarction. CMR = cardiac magnetic resonance. CV = cardiovascular. HF = heart failure. LVEDV = left ventricular end-diastolic volume. LVEF = left ventricular ejection fraction. LVESV = left ventricular end systolic volume. LVR = left ventricular remodeling. PGC-1α = peroxisome proliferator-activated receptor-gamma coactivator-1alpha VA = ventricular arrythmia.

Due to the variability of diagnostic tests and the lack of well-established criteria, multiple quantitative cut-off points have been proposed to define LVR, in some cases arbitrarily [61]. The most widely accepted definition is a >20% increase in LVEDV, which was first described by ventriculography in 1986 [17,66] and has been associated with adverse clinical outcomes [67].

### 4.2. Diagnosis of Post-AMI LVR

After the initial definition of LVR by ventriculography [66], subsequent studies primarily used echocardiography (Table 1). Bolognese et al. also used a > 20% increase in LVEDV as the cut-off, since it clearly exceeds intra-observer measurement variability as determined by the upper limit of the 95% confidence interval of the change (%Δ) in LVEDV [17]. Later studies linked this definition of LVR to an increased risk of hospitalization for HF [67]. In any case, other echocardiographic studies have used different cut-off points [74], highlighting the lack of uniformity and standardization in the research.

Regarding CMR-based studies (Table 1), several collaborative efforts have sought to unify criteria, taking advantage of CMR’s higher reproducibility and reliability. Bulluck et al. assessed LVR using lower thresholds, suggesting a 12% increase for both LVEDV and LVESV. These results were intended to predict a long-term LVEF < 50% but were not correlated with clinical events, and the sample size was small (*n* = 40) [76]. In a subsequent prospective study of 285 patients, the same authors confirmed that a ≥12% increase in both LVEDV and LVESV was associated with worse clinical outcomes, especially in the group with a concomitant ≥12% increase in both LVEDV and LVESV [79]. Reindl et al. studied 224 patients to determine the optimal cut-off value for predicting major events and proposed a 10% increase in LVEDV as the threshold [15]. Rodriguez-Palomares et al. suggested a 15% relative increase in LVEDV and a concomitant 3% relative decrease in LVEF as the cut-offs that best predicted clinical events such as CV mortality, admission for HF, or ventricular arrhythmia, in a sample of 374 patients with STEMI. These studies suggest that CMR, due to its higher spatial resolution and reproducibility, can predict an adverse prognosis even when smaller changes occur in LV volumes and LVEF.

In contrast to adverse LVR, many patients experience reverse LVR, characterized by decreased LV volumes and improved LVEF in the months following AMI. Definitions of reverse LVR also vary considerably regarding the metrics and thresholds used, although it is well established that it confers a more favorable prognosis [87,88,89].

As part of the diagnostic work-up, all AMI patients should undergo baseline imaging during the index admission (≤7 days post-AMI and before hospital discharge), preferably by echocardiography, or by CMR in complex or inconclusive cases [7,8]. Follow-up imaging during the chronic phase (3–6 months post-AMI) is recommended for patients at higher risk of LVR and/or with reduced LVEF. An increase of >20% in LVEDV is a reasonable echocardiographic threshold for defining LVR, whereas an increase of >10–15% in CMR-derived LVEDV may suffice to identify LVR, particularly when accompanied by a concomitant rise in LVESV and/or a reduction in LVEF.

### 4.3. Sex-Related Differences and Specific Populations

Women have a higher risk of mortality and HF after AMI [78,90,91,92,93]. However, evidence on sex-related differences in post-AMI LVR remains inconclusive. Some authors have reported a higher occurrence in women, while other studies have reported it in men, with some considering the remodeling process in women to be more favorable [17,94,95]. Nevertheless, recent studies report no significant differences between sexes after adjusting for confounding factors [71,78,96]. This likely indicates that sex differences in LVR and post-AMI prognosis are more attributable to variations in baseline characteristics than to intrinsic biological differences. For instance, women presenting with AMI are generally older and disproportionately affected by both traditional and non-traditional CV risk factors [97].

In elderly STEMI patients, the risk of major cardiac events and HF readmissions is markedly higher—even when LVEF reduction is modest [11,98]. Multiple causes of this worse prognosis have been described in this patient subgroup, but there is no solid evidence that this is due to a higher prevalence of adverse LVR [99]. Some studies even suggest that younger patients are more prone to adverse LVR [100]. Therefore, the mechanisms underlying the worse prognosis in elderly patients likely extend beyond structural and volumetric changes alone.

## 5. Cardiac Rehabilitation and Left Ventricular Remodeling

### 5.1. Cardiac Rehabilitation After AMI

Irrespective of LVEF and the risk of LVR, exercise-based CR is systematically recommended after AMI [7,8]. CR reduces total mortality [101], decreases the number of hospitalizations and the risk of subsequent AMI, lowers sociosanitary costs, and improves quality of life, even in contemporary cohorts [102,103].

CR is a multicomponent, multidisciplinary program that encompasses a number of interventions [104]. Perhaps the most visible part of CR is exercise training, which has traditionally been in a hospital-centered basis. However, the current trend favors individualized approaches, including ambulatory, home-based, and telerehabilitation programs for low-risk patients [105,106,107,108]. Nevertheless, CR must integrate several other interventions beyond exercise training.

AMI patients included in CR should achieve optimal CV risk factors control, facilitated by both lifestyle modification and pharmacological therapy optimization [109,110,111]. Lipid control, management of hypertension, metabolic and weight management in diabetic or overweight patients, and smoking cessation are considered core components of CR [112]. Mental health evaluation and therapy for affected individuals should be provided [113], as well as psychosocial support services [114].

Specifically in patients with mildly reduced or reduced LVEF and/or symptomatic HF after AMI, CR provides the ideal framework for initiating, up-titrating, or even de-escalating pharmacological therapy [115,116,117]. Diuretics, beta-blockers, RAAS inhibitors, sodium-glucose cotransporter-2 inhibitors (SGLT2-i), and other drugs can ameliorate symptoms, improve prognosis, and modulate LVR in patients with systolic dysfunction after AMI.

**Figure 3:**
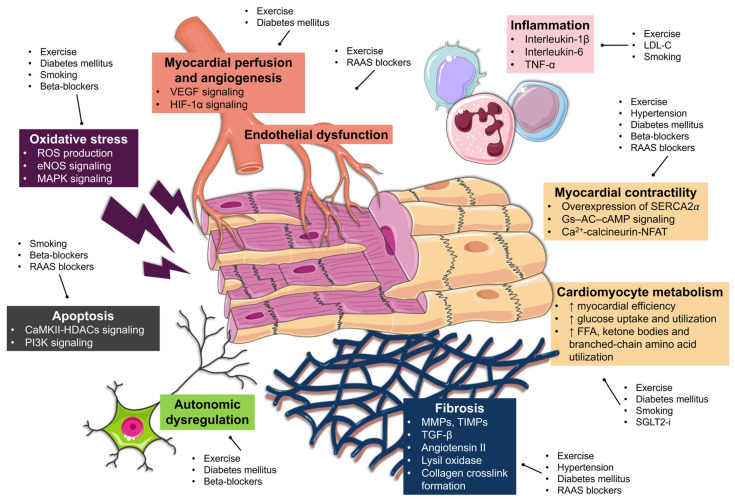
Mechanisms underlying the improvement of LVR through post-AMI Cardiac Rehabilitation. Exercise training, CV risk factors control, and optimization of pharmacological therapy can modulate post-AMI LVR. “↑” indicates increase. Abbreviations: AMI = acute myocardial infarction. CaMKII = calcium/calmodulin-dependent protein kinase II. cAMP = cyclic adenosine monophosphate. CV = cardiovascular. FFA = free fatty acids. GS-α = G-stimulatory alpha subunit. HDAC5 = histone deacetylase 5. HIF-1α = hypoxia-inducible factor 1-alpha. IL-1β = interleukin-1 beta. IL-6 = interleukin-6. LVR = left ventricular remodeling. MAPK = mitogen-activated protein kinase. MMPs = matrix metalloproteinases. NFAT = nuclear factor of activated T-cells. NOS = nitric oxide synthase. RAAS = renin–angiotensin–aldosterone system. ROS = reactive oxygen species. SERCA2a = sarcoplasmic/endoplasmic reticulum calcium ATPase 2a. SGLT2i = sodium-glucose cotransporter 2 inhibitors. TIMPs = tissue inhibitors of metalloproteinases. TNF-α = tumor necrosis factor alpha. TGF-β = transforming growth factor beta. VEGF = vascular endothelial growth factor.

Thus, exercise training, CV risk factors control, and pharmacological optimization emerge as significant factors that could modulate LVR after AMI through CR (Figure 3).

### 5.2. Exercise Training and LVR

Many decades ago, exercise training was believed to be unsafe for AMI survivors, as it was thought to trigger ventricular tachyarrhythmias and promote LV dilation [118]. However, subsequent evidence has confirmed its safety, showing that exercise does not induce adverse LVR [119,120], and can even improve LVR in the long term [121]. Current recommendations encourage AMI survivors to engage in regular physical activity, particularly moderate-to-vigorous aerobic and resistance exercise [8,122,123].

As one of the core components of CR, exercise training should begin after appropriate evaluation. Exercise testing, which can be safely performed a few days after hospital discharge if there are no contraindications, provides information regarding exercise tolerance and training intensities. Patients must then be provided with clear guidance regarding the type, duration, frequency, and target intensity of exercise, following the FITT (frequency, intensity, time, type) model [104]. Supervised in-hospital training sessions, when available, should be complemented with ambulatory sessions after education on self-monitoring methods—mainly heart rate–based and perceived exertion–based approaches (talk test, Borg RPE scale, etc.).

Exercise training shows beneficial effects on LVR, especially when started soon after discharge and maintained for longer durations [121,124]. It can improve LVEF, end-diastolic volumes, and myocardial mechanics [125,126]. These beneficial effects have also been observed in patients without significant LV systolic dysfunction [127].

Post-AMI inflammation, although necessary and beneficial to some extent [128], can be modulated by exercise training [129]. Cardiac fibrosis occurs as a physiological response to cardiomyocyte injury and death and serves to prevent wall rupture in the infarcted area. However, excessive fibrosis can expand the myocardial scar and negatively affect LVEF and LVR. Physical exercise can attenuate this exaggerated response through a complex interaction between MMPs, tissue inhibitors of matrix metalloproteinases (TIMPs), transforming growth factor-β (TGF-β), and other signaling pathways [130,131].

Exercise can improve myocardial perfusion after AMI [132,133], potentially through enhanced angiogenesis via vascular endothelial growth factor (VEGF) [37,134] and hypoxia-inducible factor-1 α (HIF-1α) signaling [135]. Additional cardioprotective effects of post-AMI exercise include reduction in oxidative stress in cardiomyocytes by modulating endothelial nitric oxide synthase (eNOS) activity and signaling [136]; increased myocardial contractility and efficiency mediated by upregulation of sarco/endoplasmic reticulum Ca2+-ATPase 2alpha (SERCA2*α*) [137]; improvement of autonomic dysregulation [138]; and enhanced myocardial energy metabolism [137].

In summary, exercise training appears to provide significant structural and functional benefits that can favorably influence post-AMI LVR.

### 5.3. CV Risk Factors Control and LVR

Poor control of CV risk factors—such as elevated blood cholesterol, high arterial pressure, and poor weight and metabolic control—can negatively influence post-AMI LVR.

Elevated blood cholesterol, and specifically low-density lipoprotein cholesterol (LDL-C), is associated with higher LV volumes [139], thromboinflammation, and adverse LVR [140]. Accordingly, statin therapy and lowering LDL-C levels have been shown to attenuate adverse post-AMI LVR [141,142,143].

Uncontrolled hypertension ultimately leads to LV dilation through several pathophysiological mechanisms [144]. An increase in cardiac afterload promotes LV hypertrophy, which ultimately leads to TGF-β and angiotensin II activation, upregulation of lysyl oxidase, collagen crosslink formation, diffuse myocardial fibrosis, and wall stiffness. Since the AMI scar already increases systolic and diastolic pressure on a weakened LV [144], further overload in cases of uncontrolled hypertension can contribute to—and in fact is associated with—post-AMI adverse LVR [145,146].

Poor metabolic control in diabetic patients can lead to diabetic cardiomyopathy, a long-term complication caused by hyperglycemia and myocardial inflammation [147]. The accumulation of advanced glycation end products (AGE) promotes collagen crosslinking and myocardial fibrosis through angiotensin II, TGF-β and TNF signaling [148]. Persistent hyperglycemia also impairs vascular and autonomic function, increases oxidative stress, and alters myocardial metabolism by enhancing the utilization of free fatty acids as a consequence of impaired glucose uptake [148].

In AMI patients, abnormal glucose metabolism has been linked to remodeling severity. Elevated glycated hemoglobin (HbA1c) levels are associated with poorer LV strain in STEMI patients [149], and high glycemic variability increases the odds of post-AMI adverse LVR [150]. Even in non-diabetic post-STEMI patients the presence of insulin resistance and dysglycemia—assessed by fasting glucose, glucose tolerance and homeostasis model assessment-estimated insulin resistance—is associated with long-term LV dilation [151].

The interplay between obesity and post-AMI LVR is complex. Obesity can induce LVR in asymptomatic individuals [152,153], especially if combined with hypertension and diabetes mellitus [154]. Conversely, weight reduction, particularly after bariatric surgery, can normalize LV volumes and mechanics [155]. Interestingly, some studies describe an “obesity paradox,” where obese patients or those with higher epicardial fat may show preserved LVEF and less adverse remodeling after AMI [156,157]. Therefore, current interventions focus on moderate (5–10%) weight loss [104], maintaining metabolic health [158], and promoting regular physical activity [159]. In fact, excess weight and glucose intolerance induced by a high-fat diet in mice after AMI attenuated the positive effects of exercise on LVR [160].

Overall, data demonstrating that CV risk factors control positively affects post-AMI LVR are scarce. Still, adherence to secondary prevention recommendations is warranted given their well-established benefits on post-AMI prognosis [7,8].

### 5.4. Smoking Habits and LVR

Smoking is a major driver of atherosclerosis and CV disease and has a direct and indirect harmful effects on cardiac morphology and function through oxidative stress, inflammation, metabolic impairment, and cell death mechanisms [161]. It has been associated with incident HF with either reduced or preserved ejection fraction [162]. Nicotine exposure has also been associated with adverse remodeling and lower LVEF in preclinical models [163,164].

Some studies have suggested that STEMI patients who were former smokers have a reduced incidence of adverse LVR [165]. Unfortunately, these studies did not account for changes in smoking status after AMI, which may act as a confounding factor. The most accepted explanation for this apparently favorable pattern is ischemic preconditioning from prior exposure to smoking.

Nevertheless, smoking cessation after AMI significantly reduces the risk of subsequent CV events [166] and mitigates tobacco-mediated cardiac metabolic and structural damage [167]. Although smoking cessation must be strongly encouraged in all post-AMI patients [168,169], further research is warranted to confirm whether smoking cessation directly improves post-AMI LVR.

### 5.5. Pharmacological Therapy Optimization and LVR

Pharmacological therapy indicated for post-AMI systolic dysfunction has generally shown benefits in terms of LVR, mainly through blood pressure control, modulation of preload and afterload, RAAS inhibition, and regulation of cardiac metabolism, inflammation, and fibrosis [16,18]. As mentioned before, these therapies can be prescribed and up-titrated within the framework of CR.

Beta-adrenergic receptor activation by local norepinephrine activity can acutely enhance cardiac output through increased chronotropy and inotropy. However, chronic beta-adrenergic activation has a detrimental effect on LVR through mitogen-activated protein kinases (MAPK) signaling, Gs–AC–cAMP signaling, Ca^2+^-calcineurinNFAT/CaMKII-HDACs signaling, and PI3K signaling [16,170]. Thus, beta-blockers are generally indicated after AMI, especially in patients with reduced (≤40%) LVEF [7]. However, their effectiveness in patients with preserved LVEF—and consequently at lower risk of adverse LVR—is currently being questioned [171].

RAAS activation also has several beneficial effects shortly after AMI, as it increases preload and cardiac contractility and maintains blood pressure and perfusion. However, in the long term angiotensin II and aldosterone promote cardiac fibrosis, cardiomyocyte apoptosis, and endothelial dysfunction [39]. Thus, neurohormonal inhibition can prevent adverse LVR and improve LVEF. Angiotensin-converting enzyme inhibitors (ACEIs), or alternatively angiotensin receptor blockers (ARBs), are indicated in high-risk AMI patients [7], such as those with LVEF ≤ 40%, since they act at different points in the RAAS cascade and inhibit adverse LVR [16]. Mineralocorticoid receptor agonists (MRAs), such as eplerenone, are recommended in symptomatic and/or diabetic post-AMI patients with LVEF ≤ 40% [172] since they improve LVR through antifibrotic effects, particularly in STEMI patients [173].

Angiotensin receptor-neprilysin inhibitors (ARNI) have proved superior to ACEIs in HF patients with LVEF ≤ 40% to reduce the risk of CV death and HF hospitalization [174]. ARNI can also promote reverse LVR in HF patients [175] and in animal models of AMI [176,177]. Although the PARADISE-MI trial did not show a significant reduction in the HF-related primary endpoint versus ramipril [178], the echocardiographic substudy revealed favorable changes in remodeling parameters [179]. Given their mechanistic profile, ARNI may offer particular benefit in STEMI patients to prevent adverse LVR [180].

SGLT2-i are a first-line therapy in patients with diabetes, HF, and chronic kidney disease, owing to their broad cardioprotective and nephroprotective effects [181]. SGLT2-i reduce the risk of CV events, especially in secondary prevention, and lower the risk of HF hospitalization and progression of renal disease in several subset of patients [181]. Structurally, SGLT2-i therapy leads to favorable LVR: in patients with diabetes or prediabetes and reduced LVEF, empagliflozin produced greater reductions in LV volumes than placebo [182], and dapagliflozin showed similar results even in non-diabetic patients [183]. Specifically after AMI, empagliflozin improved LVR in nondiabetic pigs, increasing myocardial work efficiency through a metabolic shift toward the utilization of ketone bodies, free fatty acid, and branched-chain amino acid [184]. Consistently, in the EMMY trial, AMI patients treated with empagliflozin showed greater improvement in LVEF and greater reductions in LV volumes compared to placebo [185].

However, in the EMPA-MI and DAPA-MI trials treatment with empagliflozin or dapagliflozin failed to improve the composite major adverse CV events outcome in post-AMI patients at risk of HF, although empagliflozin reduced hospitalizations for HF and dapagliflozin improved cardiometabolic outcomes [186,187]. These results provide a reasonable basis for SGLT2-i therapy in post-AMI patients with diabetes and/or reduced LVEF to improve outcomes and potentially LVR, although treatment in other post-AMI populations remains under investigation [183].

## 6. Current Clinical Management and Future Directions

### 6.1. Management of LVR During Cardiac Rehabilitation

**Figure 4:**
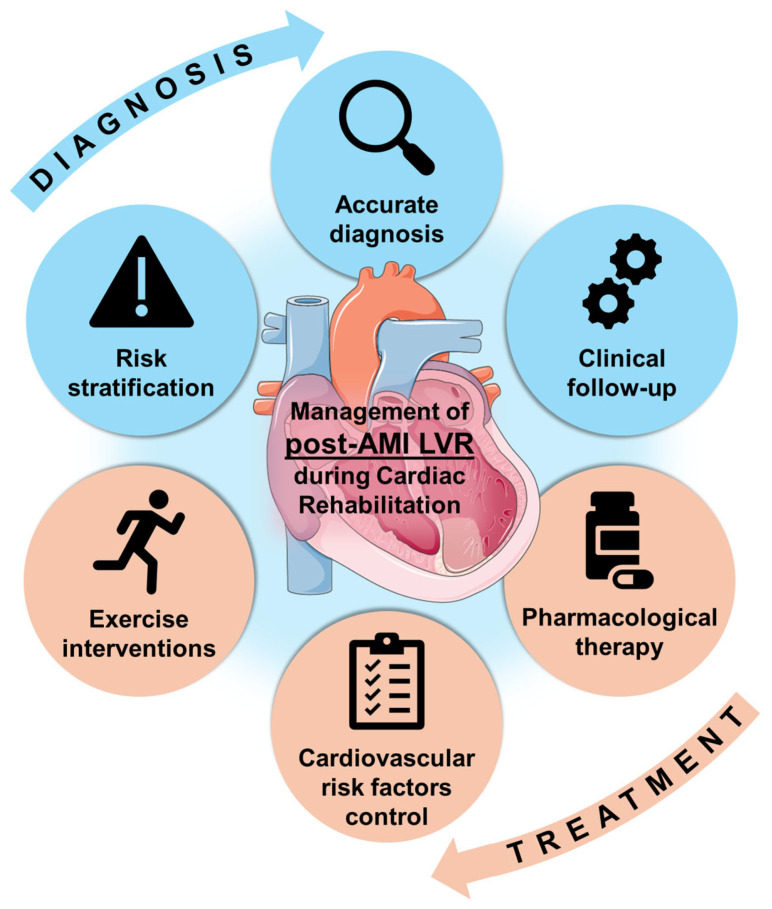
Recommendations for LVR follow-up during Cardiac Rehabilitation after AMI. Abbreviations: AMI = acute myocardial infarction. LVR = left ventricular remodeling.

Adverse LVR can be targeted during each phase of CR at both the diagnostic and therapeutic levels (Figure 4, Table 2). Phase 1 CR takes place during AMI hospitalization. Assessment of CV risk factors and early risk stratification should be performed in this phase [7,8]. Specifically, risk factors for adverse LVR should be analyzed (Table 3).

Beyond routine echocardiography before discharge [7,8], advanced CMR imaging should be considered when more detailed structural assessment is required, for instance, in those with conflicting clinical and imaging results or at high risk of post-AMI complications. Echocardiography-derived LVEF can predict which patients will benefit from an early CMR for prognostic assessment [14], and a combination of pre-discharge clinical, ECG, and echocardiographic variables can stratify the risk of CMR-detected LV thrombus [188]. Universally available ECG variables during admission can also predict long-term adverse LVR [189]. Reliable evaluation of LVEF can translate into different therapeutic management, such as the prescription of specific drugs if reduced or mildly reduced LVEF is present.

During the first months after hospital discharge, patients should be enrolled in a Phase 2 CRP. Although universal participation is ideal [7,8], selecting higher-risk patients—those with severe AMI, lower LVEF, or higher risk of adverse LVR—may be reasonable in resource-limited settings. Early enrollment in CRP after AMI appears to yield better outcomes [104].

During this phase, exercise interventions are performed according to each patient’s individual intensity thresholds [104]. Regular exercising, as well as achieving target CV risk factors control, can positively influence post-AMI LVR. However, in patients with systolic dysfunction, prescription and up-titration of HF-indicated drugs should be ensured during follow-up. Structural reassessment of LVEF and LV volumes should be performed approximately 6 to 12 weeks after discharge, especially in patients with reduced LVEF [7,8], although in several patients serial imaging can be performed at a later time. CMR imaging in this context can have implications for implantable cardioverter-defibrillator indication [190].

During Phase 2 and Phase 3 CR, patients should maintain healthy lifestyle habits, including regular exercise and adequate CV risk factors control. Outpatient follow-up visits should aim to detect risk factors for adverse LVR (Table 3), especially in patients with suboptimal treatment, i.e., those without previous indication for HF-related pharmacological therapy. For instance, serial N-terminal pro-B-type natriuretic peptide (NT-proBNP) testing can identify patients at higher risk of MACE [191], as well as sequential CMR imaging [12].

**Table 3:** Risk factors for LVR after AMI.

Categories	Risk Factor	Comments	References
Clinical factors	Age	Younger patients are more likely to experience adverse LVR, although elderly patients show a higher risk of incident HF across the full spectrum of LVEF ranges.	[11,100]
	Gender	Women have an increased risk of adverse LVR, although this association appears to be mediated by comorbidities and CV risk factors.	[78]
	Hypertension	Uncontrolled hypertension increases the risk of adverse LVR.	[145,146]
	Diabetes mellitus	Poor glycemic control is associated with adverse LVR.	[149,150,151]
	Chronic kidney disease	AMI patients with chronic kidney disease show increased risk of adverse LVR.	[59,60]
	Infarct location	Anterior location is associated with increased area at risk, larger infarct size, and adverse LVR.	[192]
	ECG parameters	Parameters such as the number of leads with Q waves and residual ST-segment elevation >1 mm have been associated with reduced LVEF, higher LV volumes, and increased infarct size during follow-up.	[189]
Imaging factors	LVEF	Although recovery from systolic dysfunction is possible, patients with initially lower LVEF have an increased risk of long-term reduced LVEF and higher LV volumes, which increases HF-related MACE.	[12,14]
	Myocardial strain	CMR-derived longitudinal and circumferential global strain, as well as strain in remote myocardium, predict adverse LVR and MACE after AMI.	[69,193,194,195]
	LVEDV and LVESV	More dilated LV volumes after AMI are associated with an increased risk of adverse LVR during follow-up.	[75,196]
	Infarct size	Larger infarct size predicts long-term risk of adverse LVR.	[197]
	MVO	Early detection of CMR-derived MVO is associated with adverse LVR and MACE. Long-term persistence of MVO is also associated with adverse structural outcomes.	[197,198,199]
Biomarkers	NT-proBNP	Higher NT-proBNP values are correlated with adverse LVR and can stratify the long-term risk of HF-related MACE.	[191,200]
	High-sensitivity troponin	Higher high-sensitivity troponin levels during admission predict lower LVEF and more extensive infarct size at long-term follow-up. Elevated levels are also associated with incident HF after AMI.	[200,201]
	sST2	Elevated sST2 levels after AMI are associated with more extensive infarctions, decreased LVEF, and higher LV volumes at follow-up.	[202]

Abbreviations: AMI = acute myocardial infarction. CMR = cardiac magnetic resonance. CV = cardiovascular. HF = heart failure. LV = left ventricular. LVEDV = left ventricular end-diastolic volume. LVEF = left ventricular ejection fraction. LVESV = left ventricular end systolic volume. LVR = left ventricular remodeling. MACE = major adverse cardiac events. MVO = microvascular obstruction. NT-proBNP = N-terminal pro-brain natriuretic peptide. sST2 = soluble suppression of tumorigenicity-2.

### 6.2. Future Directions

As the first step in the management of adverse LVR after an AMI, improved diagnostic approaches to confirm current or future LVR are being developed. Advanced echocardiography—including three-dimensional, contrast-enhanced, and strain analyses—can enhance the accuracy and reproducibility of LVEF measurements and improve risk stratification [18,203]. Nuclear molecular imaging can provide valuable information regarding several pathophysiological processes in the LVR process, such as inflammation, angiogenesis, fibrosis, and cardiac sympathetic activity [204]. However, CMR stands out as the most comprehensive imaging technique, offering detailed structural assessment, prognostic information, therapeutic guidance, and evaluation of novel interventions [205]. Determining which patients will benefit from a more detailed, CMR-based structural evaluation after AMI is likely one of the most relevant challenges in advancing precision medicine in this field [14,188,189,206].

From a therapeutic approach, novel strategies are being developed to modulate post-AMI adverse LVR. Inflammation modulation has shown positive results in animal models and AMI patients, although they have not yet been implemented in clinical practice [207]. Bone marrow-derived mesenchymal stem cell therapy and CD34+ cell transplantation could help in myocardial scar regeneration and neo-angiogenesis [208]. Non-coding RNA, protein and gene therapies can also modulate several pathways of the LVR process, although full implementation will require identification of specific targets in the complex process of post-AMI myocardial scarring [18]. As an example, the HF-REVERT trial will evaluate whether CDR132L, a synthetic antisense oligonucleotide that inhibits microRNA-132 (miR-132), can prevent or reverse adverse LVR after AMI [209]. In parallel, innovations in drug delivery—such as sustained-release platforms for MMP-9 inhibition—are opening the way to novel pharmacological strategies [210]. Lastly, surgical or transcatheter interventions to correct LV geometry could improve long-term LVR in selected patients [18].

In summary, future developments are likely to improve the detection of adverse LVR through more precise echocardiographic assessment and broader availability of advanced imaging techniques such as CMR. These advances may help identify patients at higher risk of LVR who could benefit from targeted therapies currently under investigation.

## 7. Conclusions

Adverse LVR affects a substantial proportion of post-AMI patients even in contemporary clinical practice. Although several criteria exist for LVR definition, serial imaging remains necessary for its accurate diagnosis, and CMR provides a more reliable assessment of LV volumes and systolic function. CR can favorably influence LVR not only through structured exercise interventions, but also by ensuring optimal control of CV risk factors and appropriate prescription and titration of HF-related pharmacological therapies. The identification of individuals at higher risk of adverse LVR should trigger an in-depth investigation of the structural consequences of AMI, to enable current and emerging therapies specifically targeted at modulating the LVR process.

## Figures and Tables

**Table 2:** Recommendations for management of LVR after AMI during Cardiac Rehabilitation.

**Phase 1 CRP-Admission for AMI**	
Diagnosis	Early risk stratification. Evaluate CV risk factors. Assessment of structural repercussions of AMI (LVEF, LVEDV, LVESV) *.
Treatment	Therapeutic planning for CV risk factors control. Initiation of low-intensity physical activity. Initiation of targeted therapy if LV dysfunction is present or there is risk of adverse LVR.
**Phase 2 CRP-First months after discharge**	
Diagnosis	Individualized clinical follow-up according to patient risk. Reassessment of structural repercussions of AMI (LVEF, LVEDV, LVESV) in the chronic phase *.
Treatment	Aim for achievement of CV risk factors goals. Exercise testing and exercise interventions (in-hospital and/or ambulatory) at moderate- to high-intensity levels. Optimization and up-titration of targeted therapies for LV dysfunction or LVR.
**Phase 3 CRP-Long-term follow-up**	
Diagnosis	Reassess achievement of CV risk factors goals. Follow-up (clinical, biomarkers, imaging *) for LVR monitoring.
Treatment	Maintenance of regular exercise training. Ensure therapeutic adherence (including lifestyle habits and pharmacological therapy).

* Advanced imaging (e.g., CMR) and a more detailed structural evaluation should be considered according to local availability, and especially in high-risk cases or when conflicting clinical/imaging results are present. Abbreviations: AMI = acute myocardial infarction. CMR = cardiac magnetic resonance. CRP = cardiac rehabilitation program. CV = cardiovascular. LV = left ventricular. LVEDV = left ventricular end-diastolic volume. LVEF = left ventricular ejection fraction. LVESV = left ventricular end systolic volume. LVR = left ventricular remodeling.

## Data Availability

No new data were created or analyzed in this study. Data sharing is not applicable to this article.

## Abbreviations

The following abbreviations are used in this manuscript:

AGE	Advanced glycation end products
AMI	Acute myocardial infarction
ARB	Angiotensin receptor blocker
ACEI	Angiotensin-converting enzyme inhibitor
ARNI	Angiotensin receptor–neprilysin inhibitor
BMI	Body mass index
CMR	Cardiac magnetic resonance
CR	Cardiac rehabilitation
CRP	Cardiac rehabilitation program
CV	Cardiovascular
ECG	Electrocardiogram
eNOS	Endothelial nitric oxide synthase
FITT	Frequency, intensity, time, type
HF	Heart failure
HIF-1α	Hypoxia-inducible factor-1 alpha
HbA1c	Glycated hemoglobin
LV	Left ventricle/Left ventricular
LVEF	Left ventricular ejection fraction
LVEDV	Left ventricular end-diastolic volume
LVESV	Left ventricular end-systolic volume
LVR	Left ventricular remodeling
MACE	Major adverse cardiac events
MAPK	Mitogen-activated protein kinase
MMP	Matrix metalloproteinase
MRA	Mineralocorticoid receptor antagonist
MVO	Microvascular obstruction
NT-proBNP	N-terminal pro-brain natriuretic peptide
PI3K	Phosphoinositide 3-kinase
RAAS	Renin–angiotensin–aldosterone system
RPE	Rating of perceived exertion
SERCA2α	Sarco/endoplasmic reticulum Ca^2+^-ATPase 2 alpha
SGLT2-i	Sodium–glucose cotransporter-2 inhibitor
sST2	Soluble suppression of tumorigenicity-2
STEMI	ST-elevation myocardial infarction
TGF-β	Transforming growth factor-beta
TIMP	Tissue inhibitor of metalloproteinases
TNF	Tumor necrosis factor

## References

[1] Martin S.S., Aday A.W., Allen N.B., Almarzooq Z.I., Anderson C.A.M., Arora P., Avery C.L., Baker-Smith C.M., Bansal N., Beaton A.Z. (2025). 2025 Heart Disease and Stroke Statistics: A Report of US and Global Data from the American Heart Association. Circulation.

[2] Cardiovascular Diseases (CVDs). https://www.who.int/news-room/fact-sheets/detail/cardiovascular-diseases-(cvds).

[3] Nabel E.G., Braunwald E. (2012). A Tale of Coronary Artery Disease and Myocardial Infarction. N. Engl. J. Med..

[4] McLaren J., De Alencar J.N., Aslanger E.K., Meyers H.P., Smith S.W. (2024). From ST-Segment Elevation MI to Occlusion MI. JACC Adv..

[5] Hyland S.J., Eaton R.E., Max M.E., Egbert S.B., Wong S.A., Blais D.M. (2025). Pharmacotherapy of Acute ST-Elevation Myocardial Infarction and the Pharmacist’s Role, Part 1: Patient Presentation through Revascularization. Am. J. Health Syst. Pharm..

[6] Hyland S.J., Max M.E., Eaton R.E., Wong S.A., Egbert S.B., Blais D.M. (2025). Pharmacotherapy of Acute ST-Elevation Myocardial Infarction and the Pharmacist’s Role, Part 2: Complications, Post-Revascularization Care, and Quality Improvement. Am. J. Health Syst. Pharm..

[7] Rao S.V., O’Donoghue M.L., Ruel M., Rab T., Tamis-Holland J.E., Alexander J.H., Baber U., Baker H., Cohen M.G., Cruz-Ruiz M. (2025). 2025 ACC/AHA/ACEP/NAEMSP/SCAI Guideline for the Management of Patients with Acute Coronary Syndromes: A Report of the American College of Cardiology/American Heart Association Joint Committee on Clinical Practice Guidelines. Circulation.

[8] Byrne R.A., Rossello X., Coughlan J.J., Barbato E., Berry C., Chieffo A., Claeys M.J., Dan G.-A., Dweck M.R., Galbraith M. (2023). 2023 ESC Guidelines for the Management of Acute Coronary Syndromes. Eur. Heart J..

[9] Redfern J., Gallagher R., O’Neil A., Grace S.L., Bauman A., Jennings G., Brieger D., Briffa T. (2022). Historical Context of Cardiac Rehabilitation: Learning from the Past to Move to the Future. Front. Cardiovasc. Med..

[10] Bahit M.C., Kochar A., Granger C.B. (2018). Post-Myocardial Infarction Heart Failure. JACC Heart Fail..

[11] Marcos-Garcés V., Merenciano-Gonzalez H., Gavara J., Gabaldon-Pérez A., Lopez-Lereu M.P., Monmeneu J.V., Nuñez J., Perez N., Rios-Navarro C., De Dios E. (2023). MRI Investigation of the Differential Impact of Left Ventricular Ejection Fraction after Myocardial Infarction in Elderly vs. Non-Elderly Patients to Predict Readmission for Heart Failure. J. Magn. Reson. Imaging.

[12] Gavara J., Marcos-Garces V., Lopez-Lereu M.P., Monmeneu J.V., Rios-Navarro C., de Dios E., Perez N., Merenciano H., Gabaldon A., Cànoves J. (2022). Magnetic Resonance Assessment of Left Ventricular Ejection Fraction at Any Time Post-Infarction for Prediction of Subsequent Events in a Large Multicenter STEMI Registry. J. Magn. Reson. Imaging.

[13] Marcos-Garcés V., Perez N., Gavara J., Lopez-Lereu M.P., Monmeneu J.V., Rios-Navarro C., de Dios E., Merenciano-González H., Gabaldon-Pérez A., Cànoves J. (2022). Risk Score for Early Risk Prediction by Cardiac Magnetic Resonance after Acute Myocardial Infarction. Int. J. Cardiol..

[14] Marcos-Garces V., Gavara J., Lopez-Lereu M.P., Monmeneu J.V., Rios-Navarro C., de Dios E., Perez N., Cànoves J., Gonzalez J., Minana G. (2020). Ejection Fraction by Echocardiography for a Selective Use of Magnetic Resonance After Infarction. Circ. Cardiovasc. Imaging.

[15] Reindl M., Reinstadler S.J., Tiller C., Feistritzer H.-J., Kofler M., Brix A., Mayr A., Klug G., Metzler B. (2019). Prognosis-Based Definition of Left Ventricular Remodeling after ST-Elevation Myocardial Infarction. Eur. Radiol..

[16] Zhao W., Zhao J., Rong J. (2020). Pharmacological Modulation of Cardiac Remodeling after Myocardial Infarction. Oxid. Med. Cell. Longev..

[17] Bolognese L., Neskovic A.N., Parodi G., Cerisano G., Buonamici P., Santoro G.M., Antoniucci D. (2002). Left Ventricular Remodeling After Primary Coronary Angioplasty: Patterns of Left Ventricular Dilation and Long-Term Prognostic Implications. Circulation.

[18] Frantz S., Hundertmark M.J., Schulz-Menger J., Bengel F.M., Bauersachs J. (2022). Left Ventricular Remodelling Post-Myocardial Infarction: Pathophysiology, Imaging, and Novel Therapies. Eur. Heart J..

[19] Cohn J.N., Ferrari R., Sharpe N. (2000). Cardiac Remodeling—Concepts and Clinical Implications: A Consensus Paper from an International Forum on Cardiac Remodeling. J. Am. Coll. Cardiol..

[20] Curley D., Lavin Plaza B., Shah A.M., Botnar R.M. (2018). Molecular Imaging of Cardiac Remodelling After Myocardial Infarction. Basic Res. Cardiol..

[21] Ríos-Navarro C., Gavara J., De Dios E., Pérez-Solé N., Molina-García T., Marcos-Garcés V., Ruiz-Saurí A., Bayés-Genís A., Carrión-Valero F., Chorro F.J. (2024). Effect of Serum from Patients with ST-Segment Elevation Myocardial Infarction on Endothelial Cells. Rev. Esp. Cardiol. Engl. Ed..

[22] Ortega M., Molina-García T., Gavara J., De Dios E., Pérez-Solé N., Marcos-Garcés V., Chorro F.J., Rios-Navarro C., Ruiz-Sauri A., Bodi V. (2023). Novel Targets Regulating the Role of Endothelial Cells and Angiogenesis after Infarction: A RNA Sequencing Analysis. Int. J. Mol. Sci..

[23] Ríos-Navarro C., Gavara J., Núñez J., Revuelta-López E., Monmeneu J.V., López-Lereu M.P., De Dios E., Pérez-Solé N., Vila J.M., Oltra R. (2022). EpCAM and Microvascular Obstruction in Patients with STEMI: A Cardiac Magnetic Resonance Study. Rev. Esp. Cardiol. Engl. Ed..

[24] De Dios E., Rios-Navarro C., Pérez-Solé N., Gavara J., Marcos-Garcés V., Forteza M.J., Oltra R., Vila J.M., Chorro F.J., Bodi V. (2021). Overexpression of Genes Involved in Lymphocyte Activation and Regulation Are Associated with Reduced CRM-Derived Cardiac Remodelling after STEMI. Int. Immunopharmacol..

[25] Frangogiannis N.G. (2014). The Inflammatory Response in Myocardial Injury, Repair and Remodelling. Nat. Rev. Cardiol..

[26] De Dios E., Forteza M.J., Perez-Sole N., Molina-Garcia T., Gavara J., Marcos-Garces V., Jimenez-Navarro M., Ruiz-Sauri A., Rios-Navarro C., Bodi V. (2025). Temporal and Spatial Dynamics in the Regulation of Myocardial Metabolism During the Ischemia-Reperfusion Process. Int. J. Mol. Sci..

[27] Haque Z.K., Wang D.-Z. (2017). How Cardiomyocytes Sense Pathophysiological Stresses for Cardiac Remodeling. Cell. Mol. Life Sci..

[28] Blázquez-Bujeda Á., Ortega M., De Dios E., Gavara J., Perez-Solé N., Molina-Garcia T., Marcos-Garcés V., Diaz A., Chorro F.J., Rios-Navarro C. (2023). Changes in the Extracellular Matrix at Microvascular Obstruction Area after Reperfused Myocardial Infarction: A Morphometric Study. Ann. Anat. Anat. Anz..

[29] Ortega M., Ríos-Navarro C., Gavara J., De Dios E., Perez-Solé N., Marcos-Garcés V., Ferrández-Izquierdo A., Bodí V., Ruiz-Saurí A. (2022). Meta-Analysis of Extracellular Matrix Dynamics after Myocardial Infarction Using RNA-Sequencing Transcriptomic Database. Int. J. Mol. Sci..

[30] Rios-Navarro C., Ortega M., Marcos-Garces V., Gavara J., De Dios E., Perez-Sole N., Chorro F.J., Bodi V., Ruiz-Sauri A. (2020). Interstitial Changes after Reperfused Myocardial Infarction in Swine: Morphometric and Genetic Analysis. BMC Vet. Res..

[31] Ortega M., Fábrega-García M.M., Molina-García T., Gavara J., De Dios E., Pérez-Solé N., Marcos-Garcés V., Padilla-Esquivel J.J., Diaz A., Martinez-Dolz L. (2024). Novel Fibrillar and Non-Fibrillar Collagens Involved in Fibrotic Scar Formation after Myocardial Infarction. Int. J. Mol. Sci..

[32] Frangogiannis N.G. (2017). The Extracellular Matrix in Myocardial Injury, Repair, and Remodeling. J. Clin. Investig..

[33] Ross Stewart K.M., Walker S.L., Baker A.H., Riley P.R., Brittan M. (2021). Hooked on Heart Regeneration: The Zebrafish Guide to Recovery. Cardiovasc. Res..

[34] Marcos-Garcés V., Rios-Navarro C., Gómez-Torres F., Gavara J., de Dios E., Diaz A., Miñana G., Chorro F.J., Bodi V., Ruiz-Sauri A. (2022). Fourier Analysis of Collagen Bundle Orientation in Myocardial Infarction Scars. Histochem. Cell Biol..

[35] Holmes J.W., Laksman Z., Gepstein L. (2016). Making Better Scar: Emerging Approaches for Modifying Mechanical and Electrical Properties Following Infarction and Ablation. Prog. Biophys. Mol. Biol..

[36] Wu X., Reboll M.R., Korf-Klingebiel M., Wollert K.C. (2021). Angiogenesis after Acute Myocardial Infarction. Cardiovasc. Res..

[37] Ríos-Navarro C., Hueso L., Díaz A., Marcos-Garcés V., Bonanad C., Ruiz-Sauri A., Vila J.M., Sanz M.J., Chorro F.J., Piqueras L. (2021). Role of Antiangiogenic VEGF-A165b in Angiogenesis and Systolic Function after Reperfused Myocardial Infarction. Rev. Esp. Cardiol. Engl. Ed..

[38] Sutton M.G.S.J., Sharpe N. (2000). Left Ventricular Remodeling After Myocardial Infarction: Pathophysiology and Therapy. Circulation.

[39] Leancă S.A., Crișu D., Petriș A.O., Afrăsânie I., Genes A., Costache A.D., Tesloianu D.N., Costache I.I. (2022). Left Ventricular Remodeling after Myocardial Infarction: From Physiopathology to Treatment. Life.

[40] Ferrari R., Malagù M., Biscaglia S., Fucili A., Rizzo P. (2016). Remodelling after an Infarct: Crosstalk between Life and Death. Cardiology.

[41] Yalta K., Yilmaz M.B., Yalta T., Palabiyik O., Taylan G., Zorkun C. (2020). Late Versus Early Myocardial Remodeling After Acute Myocardial Infarction: A Comparative Review on Mechanistic Insights and Clinical Implications. J. Cardiovasc. Pharmacol. Ther..

[42] Tsuda T. (2021). Clinical Assessment of Ventricular Wall Stress in Understanding Compensatory Hypertrophic Response and Maladaptive Ventricular Remodeling. J. Cardiovasc. Dev. Dis..

[43] Zhong L., Su Y., Yeo S.-Y., Tan R.-S., Ghista D.N., Kassab G. (2009). Left Ventricular Regional Wall Curvedness and Wall Stress in Patients with Ischemic Dilated Cardiomyopathy. Am. J. Physiol. Heart Circ. Physiol..

[44] Heusch G., Libby P., Gersh B., Yellon D., Böhm M., Lopaschuk G., Opie L. (2014). Cardiovascular Remodelling in Coronary Artery Disease and Heart Failure. Lancet.

[45] Richardson W.J., Holmes J.W. (2015). Why Is Infarct Expansion Such an Elusive Therapeutic Target?. J. Cardiovasc. Transl. Res..

[46] Amin P., Singh M., Singh K. (2011). β-Adrenergic Receptor-Stimulated Cardiac Myocyte Apoptosis: Role of β 1 Integrins. J. Signal Transduct..

[47] Giannoni A., Emdin M., Bramanti F., Iudice G., Francis D.P., Barsotti A., Piepoli M., Passino C. (2009). Combined Increased Chemosensitivity to Hypoxia and Hypercapnia as a Prognosticator in Heart Failure. J. Am. Coll. Cardiol..

[48] Haryono A., Ramadhiani R., Ryanto G.R.T., Emoto N. (2022). Endothelin and the Cardiovascular System: The Long Journey and Where We Are Going. Biology.

[49] Olivier A., Girerd N., Michel J., Ketelslegers J., Fay R., Vincent J., Bramlage P., Pitt B., Zannad F., Rossignol P. (2017). Combined Baseline and One-Month Changes in Big Endothelin-1 and Brain Natriuretic Peptide Plasma Concentrations Predict Clinical Outcomes in Patients with Left Ventricular Dysfunction after Acute Myocardial Infarction: Insights from the Eplerenone Post-Acute Myocardial Infarction Heart Failure Efficacy and Survival Study (EPHESUS) Study. Int. J. Cardiol..

[50] Horio T., Nishikimi T., Yoshihara F., Matsuo H., Takishita S., Kangawa K. (2000). Inhibitory Regulation of Hypertrophy by Endogenous Atrial Natriuretic Peptide in Cultured Cardiac Myocytes. Hypertension.

[51] Heidenreich P.A., Bozkurt B., Aguilar D., Allen L.A., Byun J.J., Colvin M.M., Deswal A., Drazner M.H., Dunlay S.M., Evers L.R. (2022). 2022 AHA/ACC/HFSA Guideline for the Management of Heart Failure: A Report of the American College of Cardiology/American Heart Association Joint Committee on Clinical Practice Guidelines. Circulation.

[52] McDonagh T.A., Metra M., Adamo M., Gardner R.S., Baumbach A., Böhm M., Burri H., Butler J., Čelutkienė J., Chioncel O. (2021). 2021 ESC Guidelines for the Diagnosis and Treatment of Acute and Chronic Heart Failure. Eur. Heart J..

[53] Frangogiannis N.G., Kovacic J.C. (2020). Extracellular Matrix in Ischemic Heart Disease, Part 4/4. J. Am. Coll. Cardiol..

[54] Li W. (2020). Biomechanics of Infarcted Left Ventricle: A Review of Modelling. Biomed. Eng. Lett..

[55] Nian M., Lee P., Khaper N., Liu P. (2004). Inflammatory Cytokines and Postmyocardial Infarction Remodeling. Circ. Res..

[56] Heusch G. (2020). Myocardial Ischaemia–Reperfusion Injury and Cardioprotection in Perspective. Nat. Rev. Cardiol..

[57] Jiang M., Xie X., Cao F., Wang Y. (2021). Mitochondrial Metabolism in Myocardial Remodeling and Mechanical Unloading: Implications for Ischemic Heart Disease. Front. Cardiovasc. Med..

[58] Cadenas S. (2018). ROS and Redox Signaling in Myocardial Ischemia-Reperfusion Injury and Cardioprotection. Free Radic. Biol. Med..

[59] Naito K., Anzai T., Yoshikawa T., Anzai A., Kaneko H., Kohno T., Takahashi T., Kawamura A., Ogawa S. (2008). Impact of Chronic Kidney Disease on Postinfarction Inflammation, Oxidative Stress, and Left Ventricular Remodeling. J. Card. Fail..

[60] Chiang C.-Y., Huang S.-C., Chen M., Shih J.-Y., Hong C.-S., Wu N.-C., Ho C.-H., Wu C.C., Chen Z.-C., Chang W.-T. (2021). Effects of Renal Impairment on Cardiac Remodeling and Clinical Outcomes after Myocardial Infarction. Int. J. Med. Sci..

[61] Legallois D., Hodzic A., Alexandre J., Dolladille C., Saloux E., Manrique A., Roule V., Labombarda F., Milliez P., Beygui F. (2022). Definition of Left Ventricular Remodelling Following ST-Elevation Myocardial Infarction: A Systematic Review of Cardiac Magnetic Resonance Studies in the Past Decade. Heart Fail. Rev..

[62] Grothues F., Smith G.C., Moon J.C.C., Bellenger N.G., Collins P., Klein H.U., Pennell D.J. (2002). Comparison of Interstudy Reproducibility of Cardiovascular Magnetic Resonance with Two-Dimensional Echocardiography in Normal Subjects and in Patients with Heart Failure or Left Ventricular Hypertrophy. Am. J. Cardiol..

[63] Schulz-Menger J., Bluemke D.A., Bremerich J., Flamm S.D., Fogel M.A., Friedrich M.G., Kim R.J., Von Knobelsdorff-Brenkenhoff F., Kramer C.M., Pennell D.J. (2013). Standardized Image Interpretation and Post Processing in Cardiovascular Magnetic Resonance: Society for Cardiovascular Magnetic Resonance (SCMR) Board of Trustees Task Force on Standardized Post Processing. J. Cardiovasc. Magn. Reson..

[64] Klug G., Metzler B. (2013). Assessing Myocardial Recovery Following ST-Segment Elevation Myocardial Infarction: Short- and Long-Term Perspectives Using Cardiovascular Magnetic Resonance. Expert Rev. Cardiovasc. Ther..

[65] Wong D.T.L., Richardson J.D., Puri R., Nelson A.J., Bertaso A.G., Teo K.S.L., Worthley M.I., Worthley S.G. (2012). The Role of Cardiac Magnetic Resonance Imaging Following Acute Myocardial Infarction. Eur. Radiol..

[66] McKay R.G., Pfeffer M.A., Pasternak R.C., Markis J.E., Come P.C., Nakao S., Alderman J.D., Ferguson J.J., Safian R.D., Grossman W. (1986). Left Ventricular Remodeling after Myocardial Infarction: A Corollary to Infarct Expansion. Circulation.

[67] van der Bijl P., Abou R., Goedemans L., Gersh B.J., Holmes D.R., Ajmone Marsan N., Delgado V., Bax J.J. (2020). Left Ventricular Post-Infarct Remodeling: Implications for Systolic Function Improvement and Outcomes in the Modern Era. JACC Heart Fail..

[68] Tardif J.-C., O’Meara E., Komajda M., Böhm M., Borer J.S., Ford I., Tavazzi L., Swedberg K. (2011). Effects of Selective Heart Rate Reduction with Ivabradine on Left Ventricular Remodelling and Function: Results from the SHIFT Echocardiography Substudy. Eur. Heart J..

[69] Gavara J., Rodriguez-Palomares J.F., Rios-Navarro C., Valente F., Monmeneu J.V., Lopez-Lereu M.P., Ferreira-Gonzalez I., Garcia Del Blanco B., Otaegui I., Canoves J. (2021). Longitudinal Strain in Remote Non-Infarcted Myocardium by Tissue Tracking CMR: Characterization, Dynamics, Structural and Prognostic Implications. Int. J. Cardiovasc. Imaging.

[70] Bière L., Donal E., Jacquier A., Croisille P., Genée O., Christiaens L., Prunier F., Gueret P., Boyer L., Furber A. (2016). A New Look at Left Ventricular Remodeling Definition by Cardiac Imaging. Int. J. Cardiol..

[71] Canali E., Masci P., Bogaert J., Bucciarelli Ducci C., Francone M., McAlindon E., Carbone I., Lombardi M., Desmet W., Janssens S. (2012). Impact of Gender Differences on Myocardial Salvage and Post-Ischaemic Left Ventricular Remodelling after Primary Coronary Angioplasty: New Insights from Cardiovascular Magnetic Resonance. Eur. Heart J. Cardiovasc. Imaging.

[72] Mather A.N., Fairbairn T.A., Artis N.J., Greenwood J.P., Plein S. (2011). Timing of Cardiovascular MR Imaging after Acute Myocardial Infarction: Effect on Estimates of Infarct Characteristics and Prediction of Late Ventricular Remodeling. Radiology.

[73] Mannaerts H. (2004). Early Identification of Left Ventricular Remodelling after Myocardial Infarction, Assessed by Transthoracic 3D Echocardiography. Eur. Heart J..

[74] Silveira C.F.D.S.M.P.D., Malagutte K.N.D.S., Nogueira B.F., Reis F.M., Rodrigues C.D.S.A., Rossi D.A.A., Okoshi K., Bazan R., Martin L.C., Minicucci M.F. (2021). Clinical and Echocardiographic Predictors of Left Ventricular Remodeling Following Anterior Acute Myocardial Infarction. Clinics.

[75] Logeart D., Taille Y., Derumeaux G., Gellen B., Sirol M., Galinier M., Roubille F., Georges J.-L., Trochu J.-N., Launay J.-M. (2024). Patterns of Left Ventricular Remodeling Post-Myocardial Infarction, Determinants, and Outcome. Clin. Res. Cardiol..

[76] Bulluck H., Go Y.Y., Crimi G., Ludman A.J., Rosmini S., Abdel-Gadir A., Bhuva A.N., Treibel T.A., Fontana M., Pica S. (2016). Defining Left Ventricular Remodeling Following Acute ST-Segment Elevation Myocardial Infarction Using Cardiovascular Magnetic Resonance. J. Cardiovasc. Magn. Reson..

[77] Rodriguez-Palomares J.F., Gavara J., Ferreira-González I., Valente F., Rios C., Rodríguez-García J., Bonanad C., García Del Blanco B., Miñana G., Mutuberria M. (2019). Prognostic Value of Initial Left Ventricular Remodeling in Patients with Reperfused STEMI. JACC Cardiovasc. Imaging.

[78] Alonso Tello A., Sambola A., Valente F., Sao A., Ródenas-Alesina E., Rello P., Maymi M., Barrabés J.A., Otaegui I., García Del Blanco B. (2025). Sex-Based Differences in Adverse Left Ventricular Remodelling and Clinical Outcomes after ST-Segment Elevation Myocardial Infarction. Eur. Heart J. Cardiovasc. Imaging.

[79] Bulluck H., Carberry J., Carrick D., McEntegart M., Petrie M.C., Eteiba H., Hood S., Watkins S., Lindsay M., Mahrous A. (2020). Redefining Adverse and Reverse Left Ventricular Remodeling by Cardiovascular Magnetic Resonance Following ST-Segment–Elevation Myocardial Infarction and Their Implications on Long-Term Prognosis. Circ. Cardiovasc. Imaging.

[80] Shetelig C., Limalanathan S., Hoffmann P., Seljeflot I., Gran J.M., Eritsland J., Andersen G.Ø. (2018). Association of IL-8 with Infarct Size and Clinical Outcomes in Patients with STEMI. J. Am. Coll. Cardiol..

[81] Garg P., Broadbent D.A., Swoboda P.P., Foley J.R.J., Fent G.J., Musa T.A., Ripley D.P., Erhayiem B., Dobson L.E., McDiarmid A.K. (2016). Extra-Cellular Expansion in the Normal, Non-Infarcted Myocardium Is Associated with Worsening of Regional Myocardial Function after Acute Myocardial Infarction. J. Cardiovasc. Magn. Reson..

[82] Shetye A.M., Nazir S.A., Razvi N.A., Price N., Khan J.N., Lai F.Y., Squire I.B., McCann G.P., Arnold J.R. (2017). Comparison of Global Myocardial Strain Assessed by Cardiovascular Magnetic Resonance Tagging and Feature Tracking to Infarct Size at Predicting Remodelling Following STEMI. BMC Cardiovasc. Disord..

[83] Sugano A., Seo Y., Ishizu T., Watabe H., Yamamoto M., Machino-Ohtsuka T., Takaiwa Y., Kakefuda Y., Aihara H., Fumikura Y. (2017). Value of 3-Dimensional Speckle Tracking Echocardiography in the Prediction of Microvascular Obstruction and Left Ventricular Remodeling in Patients with ST-Elevation Myocardial Infarction. Circ. J..

[84] Fabregat-Andrés Ó., Ridocci-Soriano F., Estornell-Erill J., Corbí-Pascual M., Valle-Muñoz A., Berenguer-Jofresa A., Barrabés J.A., Mata M., Monsalve M. (2015). Blood PGC-1α Concentration Predicts Myocardial Salvage and Ventricular Remodeling After ST-Segment Elevation Acute Myocardial Infarction. Rev. Esp. Cardiol. Engl. Ed..

[85] Huttin O., Mandry D., Eschalier R., Zhang L., Micard E., Odille F., Beaumont M., Fay R., Felblinger J., Camenzind E. (2016). Cardiac Remodeling Following Reperfused Acute Myocardial Infarction Is Linked to the Concomitant Evolution of Vascular Function as Assessed by Cardiovascular Magnetic Resonance. J. Cardiovasc. Magn. Reson..

[86] Eitel I., Friedenberger J., Fuernau G., Dumjahn A., Desch S., Schuler G., Thiele H. (2011). Intracoronary versus Intravenous Bolus Abciximab Application in Patients with ST-Elevation Myocardial Infarction Undergoing Primary Percutaneous Coronary Intervention: 6-Month Effects on Infarct Size and Left Ventricular Function: The Randomised Leipzig Immediate PercutaneouS Coronary Intervention Abciximab i.v. versus i.c. in ST-Elevation Myocardial Infarction Trial (LIPSIAbciximab-STEMI). Clin. Res. Cardiol..

[87] Funaro S., La Torre G., Madonna M., Galiuto L., Scara A., Labbadia A., Canali E., Mattatelli A., Fedele F., Alessandrini F. (2009). Incidence, Determinants, and Prognostic Value of Reverse Left Ventricular Remodelling after Primary Percutaneous Coronary Intervention: Results of the Acute Myocardial Infarction Contrast Imaging (AMICI) Multicenter Study. Eur. Heart J..

[88] Daubert M.A., White J.A., Al-Khalidi H.R., Velazquez E.J., Rao S.V., Crowley A.L., Zeymer U., Kasprzak J.D., Guetta V., Krucoff M.W. (2020). Cardiac Remodeling after Large ST-Elevation Myocardial Infarction in the Current Therapeutic Era. Am. Heart J..

[89] Hnat T., Veselka J., Honek J. (2022). Left Ventricular Reverse Remodelling and Its Predictors in Non-ischaemic Cardiomyopathy. ESC Heart Fail..

[90] Hao Y., Liu J., Liu J., Yang N., Smith S.C., Huo Y., Fonarow G.C., Ge J., Taubert K.A., Morgan L. (2019). Sex Differences in In-Hospital Management and Outcomes of Patients with Acute Coronary Syndrome: Findings from the CCC Project. Circulation.

[91] Aimo A., Panichella G., Barison A., Maffei S., Cameli M., Coiro S., D’Ascenzi F., Di Mario C., Liga R., Marcucci R. (2021). Sex-Related Differences in Ventricular Remodeling after Myocardial Infarction. Int. J. Cardiol..

[92] Huded C.P., Johnson M., Kravitz K., Menon V., Abdallah M., Gullett T.C., Hantz S., Ellis S.G., Podolsky S.R., Meldon S.W. (2018). 4-Step Protocol for Disparities in STEMI Care and Outcomes in Women. J. Am. Coll. Cardiol..

[93] Kosmidou I., Redfors B., Selker H.P., Thiele H., Patel M.R., Udelson J.E., Magnus Ohman E., Eitel I., Granger C.B., Maehara A. (2017). Infarct Size, Left Ventricular Function, and Prognosis in Women Compared to Men after Primary Percutaneous Coronary Intervention in ST-Segment Elevation Myocardial Infarction: Results from an Individual Patient-Level Pooled Analysis of 10 Randomized Trials. Eur. Heart J..

[94] Piro M., Della Bona R., Abbate A., Biasucci L.M., Crea F. (2010). Sex-Related Differences in Myocardial Remodeling. J. Am. Coll. Cardiol..

[95] Garber L., McAndrew T.C., Chung E.S., Stancak B., Svendsen J.H., Monteiro J., Fischer T.M., Kueffer F., Ryan T., Bax J. (2018). Predictors of Left Ventricular Remodeling After Myocardial Infarction in Patients with a Patent Infarct Related Coronary Artery After Percutaneous Coronary Intervention (from the Post-Myocardial Infarction Remodeling Prevention Therapy [PRomPT] Trial). Am. J. Cardiol..

[96] Van Der Bijl P., Abou R., Goedemans L., Gersh B.J., Holmes D.R., Ajmone Marsan N., Delgado V., Bax J.J. (2020). Left Ventricular Remodelling after ST-segment Elevation Myocardial Infarction: Sex Differences and Prognosis. ESC Heart Fail..

[97] Zilio F., Musella F., Ceriello L., Ciliberti G., Pavan D., Manes M.T., Selimi A., Scicchitano P., Iannopollo G., Albani S. (2024). Sex Differences in Patients Presenting with Acute Coronary Syndrome: A State-of-the-Art Review. Curr. Probl. Cardiol..

[98] Gabaldón-Pérez A., Marcos-Garcés V., Gavara J., López-Lereu M.P., Monmeneu J.V., Pérez N., Ríos-Navarro C., de Dios E., Merenciano-González H., Cànoves J. (2022). Prognostic Value of Cardiac Magnetic Resonance Early after ST-Segment Elevation Myocardial Infarction in Older Patients. Age Ageing.

[99] Ennezat P.V., Lamblin N., Mouquet F., Tricot O., Quandalle P., Aumegeat V., Equine O., Nugue O., Segrestin B., De Groote P. (2008). The Effect of Ageing on Cardiac Remodelling and Hospitalization for Heart Failure after an Inaugural Anterior Myocardial Infarction. Eur. Heart J..

[100] Guo R., Wang X., Guo Q., Yan Y., Gong W., Zheng W., Zhao G., Wang H., Xu L., Nie S. (2023). Cardiac Magnetic Resonance Shows Increased Adverse Ventricular Remodeling in Younger Patients after ST-Segment Elevation Myocardial Infarction. Eur. Radiol..

[101] Salzwedel A., Jensen K., Rauch B., Doherty P., Metzendorf M.-I., Hackbusch M., Völler H., Schmid J.-P., Davos C.H. (2020). Effectiveness of Comprehensive Cardiac Rehabilitation in Coronary Artery Disease Patients Treated According to Contemporary Evidence Based Medicine: Update of the Cardiac Rehabilitation Outcome Study (CROS-II). Eur. J. Prev. Cardiol..

[102] Dibben G., Faulkner J., Oldridge N., Rees K., Thompson D.R., Zwisler A.-D., Taylor R.S. (2021). Exercise-Based Cardiac Rehabilitation for Coronary Heart Disease. Cochrane Database Syst. Rev..

[103] Dibben G.O., Faulkner J., Oldridge N., Rees K., Thompson D.R., Zwisler A.-D., Taylor R.S. (2023). Exercise-Based Cardiac Rehabilitation for Coronary Heart Disease: A Meta-Analysis. Eur. Heart J..

[104] Ambrosetti M., Abreu A., Corrà U., Davos C.H., Hansen D., Frederix I., Iliou M.C., Pedretti R.F.E., Schmid J.-P., Vigorito C. (2021). Secondary Prevention through Comprehensive Cardiovascular Rehabilitation: From Knowledge to Implementation. 2020 Update. A Position Paper from the Secondary Prevention and Rehabilitation Section of the European Association of Preventive Cardiology. Eur. J. Prev. Cardiol..

[105] Scherrenberg M., Falter M., Dendale P. (2020). Cost-Effectiveness of Cardiac Telerehabilitation in Coronary Artery Disease and Heart Failure Patients: Systematic Review of Randomized Controlled Trials. Eur. Heart J. Digit. Health.

[106] Scherrenberg M., Wilhelm M., Hansen D., Völler H., Cornelissen V., Frederix I., Kemps H., Dendale P. (2021). The Future Is Now: A Call for Action for Cardiac Telerehabilitation in the COVID-19 Pandemic from the Secondary Prevention and Rehabilitation Section of the European Association of Preventive Cardiology. Eur. J. Prev. Cardiol..

[107] Golbus J.R., Lopez-Jimenez F., Barac A., Cornwell W.K., Dunn P., Forman D.E., Martin S.S., Schorr E.N., Supervia M. (2023). Digital Technologies in Cardiac Rehabilitation: A Science Advisory from the American Heart Association. Circulation.

[108] Nkonde-Price C., Reynolds K., Najem M., Yang S.-J., Batiste C., Cotter T., Lahti D., Gin N., Funahashi T. (2022). Comparison of Home-Based vs Center-Based Cardiac Rehabilitation in Hospitalization, Medication Adherence, and Risk Factor Control Among Patients with Cardiovascular Disease. JAMA Netw. Open.

[109] Bertolín-Boronat C., Merenciano-González H., Marcos-Garcés V., Martínez Mas M.L., Climent Alberola J.I., Civera J.M., Valls Reig M., Ruiz Hueso M., Castro Carmona P., Perez N. (2025). Low-Density Lipoprotein Cholesterol Reduction and Therapeutic Adherence During Cardiac Rehabilitation After Myocardial Infarction. J. Clin. Med..

[110] Bertolín-Boronat C., Merenciano-González H., Marcos-Garcés V., Martínez-Mas M.L., Climent Alberola J.I., Pérez N., López-Bueno L., Esteban-Argente M.C., Valls Reig M., Arizón Benito A. (2025). Dynamics of HDL-Cholesterol Following a Post-Myocardial Infarction Cardiac Rehabilitation Program. Rev. Cardiovasc. Med..

[111] Marcos-Garcés V., Merenciano-González H., Martínez Mas M.L., Palau P., Climent Alberola J.I., Perez N., López-Bueno L., Esteban Argente M.C., Valls Reig M., Muñoz Alcover R. (2024). Short-Course High-Intensity Statin Treatment during Admission for Myocardial Infarction and LDL-Cholesterol Reduction—Impact on Tailored Lipid-Lowering Therapy at Discharge. J. Clin. Med..

[112] Brown T.M., Pack Q.R., Aberegg E., Brewer L.C., Ford Y.R., Forman D.E., Gathright E.C., Khadanga S., Ozemek C., Thomas R.J. (2024). Core Components of Cardiac Rehabilitation Programs: 2024 Update: A Scientific Statement from the American Heart Association and the American Association of Cardiovascular and Pulmonary Rehabilitation. Circulation.

[113] Bertolín-Boronat C., Marcos-Garcés V., Merenciano-González H., Martínez Mas M.L., Climent Alberola J.I., Perez N., López-Bueno L., Esteban Argente M.C., Valls Reig M., Arizón Benito A. (2025). Depression, Anxiety, and Quality of Life in a Cardiac Rehabilitation Program Without Dedicated Mental Health Resources Post-Myocardial Infarction. J. Cardiovasc. Dev. Dis..

[114] Bush M., Evenson K.R., Aylward A., Cyr J.M., Kucharska-Newton A. (2023). Psychosocial Services Provided by Licensed Cardiac Rehabilitation Programs. Front. Rehabil. Sci..

[115] Goyal P., Gorodeski E.Z., Marcum Z.A., Forman D.E. (2019). Cardiac Rehabilitation to Optimize Medication Regimens in Heart Failure. Clin. Geriatr. Med..

[116] Bozkurt B., Fonarow G.C., Goldberg L.R., Guglin M., Josephson R.A., Forman D.E., Lin G., Lindenfeld J., O’Connor C., Panjrath G. (2021). Cardiac Rehabilitation for Patients with Heart Failure. J. Am. Coll. Cardiol..

[117] Bozkurt B. (2024). Contemporary Pharmacological Treatment and Management of Heart Failure. Nat. Rev. Cardiol..

[118] Braunwald E. (1998). Evolution of the Management of Acute Myocardial Infarction: A 20th Century Saga. Lancet.

[119] Giannuzzi P., Tavazzi L., Temporelli P.L., Corrá U., Imparato A., Gattone M., Giordano A., Sala L., Schweiger C., Malinverni C. (1993). Long-Term Physical Training and Left Ventricular Remodelling after Anterior Myocardial Infraction: Results of the Excercise in Anterior Myocardial Infraction (EAMI) Trial. J. Am. Coll. Cardiol..

[120] Dubach P., Myers J., Dziekan G., Goebbels U., Reinhart W., Vogt P., Ratti R., Muller P., Miettunen R., Buser P. (1997). Effect of Exercise Training on Myocardial Remodeling in Patients with Reduced Left Ventricular Function After Myocardial Infarction: Application of Magnetic Resonance Imaging. Circulation.

[121] Haykowsky M., Scott J., Esch B., Schopflocher D., Myers J., Paterson I., Warburton D., Jones L., Clark A.M. (2011). A Meta-Analysis of the Effects of Exercise Training on Left Ventricular Remodeling Following Myocardial Infarction: Start Early and Go Longer for Greatest Exercise Benefits on Remodeling. Trials.

[122] Vrints C., Andreotti F., Koskinas K.C., Rossello X., Adamo M., Ainslie J., Banning A.P., Budaj A., Buechel R.R., Chiariello G.A. (2024). 2024 ESC Guidelines for the Management of Chronic Coronary Syndromes. Eur. Heart J..

[123] Virani S.S., Newby L.K., Arnold S.V., Bittner V., Brewer L.C., Demeter S.H., Dixon D.L., Fearon W.F., Hess B., Johnson H.M. (2023). 2023 AHA/ACC/ACCP/ASPC/NLA/PCNA Guideline for the Management of Patients with Chronic Coronary Disease: A Report of the American Heart Association/American College of Cardiology Joint Committee on Clinical Practice Guidelines. Circulation.

[124] Zhang Y.-M., Lu Y., Tang Y., Yang D., Wu H.-F., Bian Z.-P., Xu J.-D., Gu C.-R., Wang L.-S., Chen X.-J. (2016). The Effects of Different Initiation Time of Exercise Training on Left Ventricular Remodeling and Cardiopulmonary Rehabilitation in Patients with Left Ventricular Dysfunction after Myocardial Infarction. Disabil. Rehabil..

[125] McGregor G., Stöhr E.J., Oxborough D., Kimani P., Shave R. (2018). Effect of Exercise Training on Left Ventricular Mechanics after Acute Myocardial Infarction–an Exploratory Study. Ann. Phys. Rehabil. Med..

[126] Zhang X., Mi Y., Ding M., Gao X. (2025). Effects of Exercise Training on Left Ventricular Systolic and Diastolic Function after Myocardial Infarction: Systematic Review and Meta-Analysis. Front. Cardiovasc. Med..

[127] McGregor G., Gaze D., Oxborough D., O’Driscoll J., Shave R. (2016). Reverse Left Ventricular Remodeling: Effect of Cardiac Rehabilitation Exercise Training in Myocardial Infarction Patients with Preserved Ejection Fraction. Eur. J. Phys. Rehabil. Med..

[128] Matter M.A., Paneni F., Libby P., Frantz S., Stähli B.E., Templin C., Mengozzi A., Wang Y.-J., Kündig T.M., Räber L. (2024). Inflammation in Acute Myocardial Infarction: The Good, the Bad and the Ugly. Eur. Heart J..

[129] Frodermann V., Rohde D., Courties G., Severe N., Schloss M.J., Amatullah H., McAlpine C.S., Cremer S., Hoyer F.F., Ji F. (2019). Exercise Reduces Inflammatory Cell Production and Cardiovascular Inflammation via Instruction of Hematopoietic Progenitor Cells. Nat. Med..

[130] Ma Y., Kuang Y., Bo W., Liang Q., Zhu W., Cai M., Tian Z. (2021). Exercise Training Alleviates Cardiac Fibrosis through Increasing Fibroblast Growth Factor 21 and Regulating TGF-Β1-Smad2/3-MMP2/9 Signaling in Mice with Myocardial Infarction. Int. J. Mol. Sci..

[131] Xu X., Wan W., Powers A.S., Li J., Ji L.L., Lao S., Wilson B., Erikson J.M., Zhang J.Q. (2008). Effects of Exercise Training on Cardiac Function and Myocardial Remodeling in Post Myocardial Infarction Rats. J. Mol. Cell. Cardiol..

[132] Giallauria F., Acampa W., Ricci F., Vitelli A., Torella G., Lucci R., Del Prete G., Zampella E., Assante R., Rengo G. (2013). Exercise Training Early after Acute Myocardial Infarction Reduces Stress-Induced Hypoperfusion and Improves Left Ventricular Function. Eur. J. Nucl. Med. Mol. Imaging.

[133] Giallauria F., Acampa W., Ricci F., Vitelli A., Maresca L., Mancini M., Grieco A., Gallicchio R., Xhoxhi E., Spinelli L. (2012). Effects of Exercise Training Started within 2 Weeks after Acute Myocardial Infarction on Myocardial Perfusion and Left Ventricular Function: A Gated SPECT Imaging Study. Eur. J. Prev. Cardiol..

[134] Leosco D., Rengo G., Iaccarino G., Golino L., Marchese M., Fortunato F., Zincarelli C., Sanzari E., Ciccarelli M., Galasso G. (2008). Exercise Promotes Angiogenesis and Improves β-Adrenergic Receptor Signalling in the Post-Ischaemic Failing Rat Heart. Cardiovasc. Res..

[135] Song W., Liang Q., Cai M., Tian Z. (2020). HIF-1α-induced Up-regulation of microRNA-126 Contributes to the Effectiveness of Exercise Training on Myocardial Angiogenesis in Myocardial Infarction Rats. J. Cell. Mol. Med..

[136] Van Deel E.D., Octavia Y., De Waard M.C., De Boer M., Duncker D.J. (2018). Exercise Training Has Contrasting Effects in Myocardial Infarction and Pressure Overload Due to Divergent Endothelial Nitric Oxide Synthase Regulation. Int. J. Mol. Sci..

[137] Liu S., Meng X., Li G., Gokulnath P., Wang J., Xiao J. (2022). Exercise Training after Myocardial Infarction Attenuates Dysfunctional Ventricular Remodeling and Promotes Cardiac Recovery. Rev. Cardiovasc. Med..

[138] Rodrigues B., Lira F.S., Consolim-Colombo F.M., Rocha J.A., Caperuto E.C., De Angelis K., Irigoyen M.-C. (2014). Role of Exercise Training on Autonomic Changes and Inflammatory Profile Induced by Myocardial Infarction. Mediators Inflamm..

[139] Aung N., Sanghvi M.M., Piechnik S.K., Neubauer S., Munroe P.B., Petersen S.E. (2020). The Effect of Blood Lipids on the Left Ventricle. J. Am. Coll. Cardiol..

[140] Ozawa K., Packwood W., Varlamov O., Muller M., Xie A., Wu M.D., Abraham-Fan R.-J., López J.A., Lindner J.R. (2023). Elevated LDL Cholesterol Increases Microvascular Endothelial VWF and Thromboinflammation After Myocardial Infarction. Arterioscler. Thromb. Vasc. Biol..

[141] Xiao X., Chang G., Liu J., Sun G., Liu L., Qin S., Zhang D. (2016). Simvastatin Ameliorates Ventricular Remodeling via the TGF-Β1 Signaling Pathway in Rats Following Myocardial Infarction. Mol. Med. Rep..

[142] Luo R., Sun X., Shen F., Hong B., Wang Z. (2020). Effects of High-Dose Rosuvastatin on Ventricular Remodelling and Cardiac Function in ST-Segment Elevation Myocardial Infarction. Drug Des. Devel. Ther..

[143] Buono F., Spinelli L., Giallauria F., Assante Di Panzillo E., Di Marino S., Ferrara F., Vigorito C., Trimarco B., Morisco C. (2011). Usefulness of Satisfactory Control of Low-Density Lipoprotein Cholesterol to Predict Left Ventricular Remodeling After a First ST-Elevation Myocardial Infarction Successfully Reperfused. Am. J. Cardiol..

[144] Tomek J., Bub G. (2017). Hypertension-induced Remodelling: On the Interactions of Cardiac Risk Factors. J. Physiol..

[145] Kenchaiah S., Pfeffer M.A., Sutton M.S.J., Plappert T., Rouleau J.-L., Lamas G.A., Sasson Z., Parker J.O., Geltman E.M., Solomon S.D. (2004). Effect of Antecedent Systemic Hypertension on Subsequent Left Ventricular Dilation after Acute Myocardial Infarction (from the Survival and Ventricular Enlargement Trial). Am. J. Cardiol..

[146] Richards A.M., Nicholls M.G., Troughton R.W., Lainchbury J.G., Elliott J., Frampton C., Espiner E.A., Crozier I.G., Yandle T.G., Turner J. (2002). Antecedent Hypertension and Heart Failure after Myocardial Infarction. J. Am. Coll. Cardiol..

[147] Rizza V., Tondi L., Patti A.M., Cecchi D., Lombardi M., Perone F., Ambrosetti M., Rizzo M., Cianflone D., Maranta F. (2024). Diabetic Cardiomyopathy: Pathophysiology, Imaging Assessment and Therapeutical Strategies. Int. J. Cardiol. Cardiovasc. Risk Prev..

[148] Mann C., Braunwald E., Zelniker T.A. (2025). Diabetic Cardiomyopathy Revisited. The Interplay between Diabetes and Heart Failure. Int. J. Cardiol..

[149] Guo Y., Guo Q., Guo R., Yan Y., Gong W., Zheng W., Wang H., Xu L., Wang X., Nie S. (2024). Glycemic Status and Myocardial Strain by Cardiac MRI in Patients with ST-Segment Elevation Myocardial Infarction. J. Magn. Reson. Imaging.

[150] Hanajima Y., Iwahashi N., Kirigaya J., Horii M., Minamimoto Y., Gohbara M., Abe T., Okada K., Matsuzawa Y., Kosuge M. (2023). Prognostic Importance of Glycemic Variability on Left Ventricular Reverse Remodeling after the First Episode of ST-Segment Elevation Myocardial Infarction. Cardiovasc. Diabetol..

[151] Yang C.D., Shen Y., Lu L., Ding F.H., Yang Z.K., Zhang R.Y., Shen W.F., Jin W., Wang X.Q. (2019). Insulin Resistance and Dysglycemia Are Associated with Left Ventricular Remodeling after Myocardial Infarction in Non-Diabetic Patients. Cardiovasc. Diabetol..

[152] Liu J., Li J., Pu H., He W., Zhou X., Tong N., Peng L. (2022). Cardiac Remodeling and Subclinical Left Ventricular Dysfunction in Adults with Uncomplicated Obesity: A Cardiovascular Magnetic Resonance Study. Quant. Imaging Med. Surg..

[153] Alpert M.A., Karthikeyan K., Abdullah O., Ghadban R. (2018). Obesity and Cardiac Remodeling in Adults: Mechanisms and Clinical Implications. Prog. Cardiovasc. Dis..

[154] Von Jeinsen B., Vasan R.S., McManus D.D., Mitchell G.F., Cheng S., Xanthakis V. (2020). Joint Influences of Obesity, Diabetes, and Hypertension on Indices of Ventricular Remodeling: Findings from the Community-Based Framingham Heart Study. PLoS ONE.

[155] Sorimachi H., Obokata M., Omote K., Reddy Y.N.V., Takahashi N., Koepp K.E., Ng A.C.T., Rider O.J., Borlaug B.A. (2022). Long-Term Changes in Cardiac Structure and Function Following Bariatric Surgery. J. Am. Coll. Cardiol..

[156] Mouton A.J., Flynn E.R., Moak S.P., Li X., Da Silva A.A., Wang Z., Do Carmo J.M., Hall M.E., Hall J.E. (2021). Interaction of Obesity and Hypertension on Cardiac Metabolic Remodeling and Survival Following Myocardial Infarction. J. Am. Heart Assoc..

[157] Gavara J., Merenciano-Gonzalez H., Llopis-Lorente J., Molina-Garcia T., Perez-Solé N., De Dios E., Marcos-Garces V., Monmeneu J.V., Lopez-Lereu M.P., Canoves J. (2024). Impact of Epicardial Adipose Tissue on Infarct Size and Left Ventricular Systolic Function in Patients with Anterior ST-Segment Elevation Myocardial Infarction. Diagnostics.

[158] Chew N.W.S., Kong G., Venisha S., Chin Y.H., Ng C.H., Muthiah M., Khoo C.M., Chai P., Kong W., Poh K.-K. (2022). Long-Term Prognosis of Acute Myocardial Infarction Associated with Metabolic Health and Obesity Status. Endocr. Pract..

[159] Moholdt T., Lavie C.J., Nauman J. (2018). Sustained Physical Activity, Not Weight Loss, Associated with Improved Survival in Coronary Heart Disease. J. Am. Coll. Cardiol..

[160] Peres Valgas Da Silva C., Shettigar V.K., Baer L.A., Abay E., Pinckard K.M., Vinales J., Sturgill S.L., Vidal P., Ziolo M.T., Stanford K.I. (2022). Exercise Training after Myocardial Infarction Increases Survival but Does Not Prevent Adverse Left Ventricle Remodeling and Dysfunction in High-Fat Diet Fed Mice. Life Sci..

[161] Kaplan A., Abidi E., Ghali R., Booz G.W., Kobeissy F., Zouein F.A. (2017). Functional, Cellular, and Molecular Remodeling of the Heart under Influence of Oxidative Cigarette Tobacco Smoke. Oxid. Med. Cell. Longev..

[162] Kamimura D., Yimer W.K., Mentz R.J., Shah A.M., White W.B., Blaha M.J., Oshunbade A., Hamid A., Suzuki T., Clark D.R. (2024). Cigarette Smoking, Smoking Cessation, and Heart Failure Subtypes: Insights from the Jackson Heart Study. J. Am. Heart Assoc..

[163] Savko C., Esquer C., Molinaro C., Rokaw S., Shain A.G., Jaafar F., Wright M.K., Phillips J.A., Hopkins T., Mikhail S. (2025). Myocardial Infarction Injury Is Exacerbated by Nicotine in Vape Aerosol Exposure. J. Am. Heart Assoc..

[164] Fried N.D., Oakes J.M., Whitehead A.K., Lazartigues E., Yue X., Gardner J.D. (2022). Nicotine and Novel Tobacco Products Drive Adverse Cardiac Remodeling and Dysfunction in Preclinical Studies. Front. Cardiovasc. Med..

[165] Symons R., Masci P.G., Francone M., Claus P., Barison A., Carbone I., Agati L., Galea N., Janssens S., Bogaert J. (2016). Impact of Active Smoking on Myocardial Infarction Severity in Reperfused ST-Segment Elevation Myocardial Infarction Patients: The Smoker’s Paradox Revisited. Eur. Heart J..

[166] Wu A.D., Lindson N., Hartmann-Boyce J., Wahedi A., Hajizadeh A., Theodoulou A., Thomas E.T., Lee C., Aveyard P. (2022). Smoking Cessation for Secondary Prevention of Cardiovascular Disease. Cochrane Database Syst. Rev..

[167] Aid J., Tanjeko A.T., Serré J., Eggelbusch M., Noort W., De Wit G.M.J., Van Weeghel M., Puurand M., Tepp K., Gayan-Ramirez G. (2024). Smoking Cessation Only Partially Reverses Cardiac Metabolic and Structural Remodeling in Mice. Acta Physiol..

[168] Barua R.S., Rigotti N.A., Benowitz N.L., Cummings K.M., Jazayeri M.-A., Morris P.B., Ratchford E.V., Sarna L., Stecker E.C., Wiggins B.S. (2018). 2018 ACC Expert Consensus Decision Pathway on Tobacco Cessation Treatment. J. Am. Coll. Cardiol..

[169] Nazir A., Shetty Ujjar S., Seddiki M.O., Jheinga M., Fan L. (2025). Smoking Cessation Strategies After Acute Coronary Syndrome. J. Clin. Med..

[170] Yang J., Liu Y., Fan X., Li Z., Cheng Y. (2014). A Pathway and Network Review on Beta-Adrenoceptor Signaling and Beta Blockers in Cardiac Remodeling. Heart Fail. Rev..

[171] Cataldo Miranda P., Gasevic D., Trin C., Stub D., Zoungas S., Kaye D.M., Orman Z., Eliakundu A.L., Talic S. (2025). Beta-Blocker Therapy After Myocardial Infarction. JACC Adv..

[172] Pitt B., Remme W., Zannad F., Neaton J., Martinez F., Roniker B., Bittman R., Hurley S., Kleiman J., Gatlin M. (2003). Eplerenone, a Selective Aldosterone Blocker, in Patients with Left Ventricular Dysfunction after Myocardial Infarction. N. Engl. J. Med..

[173] Toda K., Kasama S., Toyama T., Kasahara M., Kurabayashi M. (2022). Effects of Mineralocorticoid Receptor Antagonist Eplerenone on Cardiac Sympathetic Nerve Activity and Left Ventricular Remodeling after Reperfusion Therapy in Patients with First ST-Segment Elevation Myocardial Infarction. J. Nucl. Cardiol..

[174] McMurray J.J.V., Packer M., Desai A.S., Gong J., Lefkowitz M.P., Rizkala A.R., Rouleau J.L., Shi V.C., Solomon S.D., Swedberg K. (2014). Angiotensin–Neprilysin Inhibition versus Enalapril in Heart Failure. N. Engl. J. Med..

[175] Wang Y., Zhou R., Lu C., Chen Q., Xu T., Li D. (2019). Effects of the Angiotensin-Receptor Neprilysin Inhibitor on Cardiac Reverse Remodeling: Meta-Analysis. J. Am. Heart Assoc..

[176] Torrado J., Cain C., Mauro A.G., Romeo F., Ockaili R., Chau V.Q., Nestler J.A., Devarakonda T., Ghosh S., Das A. (2018). Sacubitril/Valsartan Averts Adverse Post-Infarction Ventricular Remodeling and Preserves Systolic Function in Rabbits. J. Am. Coll. Cardiol..

[177] Pfau D., Thorn S.L., Zhang J., Mikush N., Renaud J.M., Klein R., deKemp R.A., Wu X., Hu X., Sinusas A.J. (2019). Angiotensin Receptor Neprilysin Inhibitor Attenuates Myocardial Remodeling and Improves Infarct Perfusion in Experimental Heart Failure. Sci. Rep..

[178] Pfeffer M.A., Claggett B., Lewis E.F., Granger C.B., Køber L., Maggioni A.P., Mann D.L., McMurray J.J.V., Rouleau J.-L., Solomon S.D. (2021). Angiotensin Receptor–Neprilysin Inhibition in Acute Myocardial Infarction. N. Engl. J. Med..

[179] Shah A.M., Claggett B., Prasad N., Li G., Volquez M., Jering K., Cikes M., Kovacs A., Mullens W., Nicolau J.C. (2022). Impact of Sacubitril/Valsartan Compared with Ramipril on Cardiac Structure and Function After Acute Myocardial Infarction: The PARADISE-MI Echocardiographic Substudy. Circulation.

[180] Bellis A., Mauro C., Barbato E., Trimarco B., Morisco C. (2022). The PARADISE-MI Trial: A New Opportunity to Improve the Left Ventricular Remodelling in Reperfused STEMI. ESC Heart Fail..

[181] Kumar N., Kumar B., Ashique S., Yasmin S., Venkatesan K., Islam A., Ghosh S., Sahu A., Bhui U., Ansari M.Y. (2025). A Critical Review on SGLT2 Inhibitors for Diabetes Mellitus, Renal Health, and Cardiovascular Conditions. Diabetes Res. Clin. Pract..

[182] Lee M.M.Y., Brooksbank K.J.M., Wetherall K., Mangion K., Roditi G., Campbell R.T., Berry C., Chong V., Coyle L., Docherty K.F. (2021). Effect of Empagliflozin on Left Ventricular Volumes in Patients with Type 2 Diabetes, or Prediabetes, and Heart Failure with Reduced Ejection Fraction (SUGAR-DM-HF). Circulation.

[183] Acquaro M., Scelsi L., Mascolo C., Pelosi C., Greco A., Turco A., Schirinzi S., Lattanzio M., Resasco T., Mercurio V. (2025). Dapagliflozin Effects on Left Ventricular Remodeling and Filling Pressures in Heart Failure with Reduced Ejection Fraction. J. Am. Soc. Echocardiogr..

[184] Santos-Gallego C.G., Requena-Ibanez J.A., San Antonio R., Ishikawa K., Watanabe S., Picatoste B., Flores E., Garcia-Ropero A., Sanz J., Hajjar R.J. (2019). Empagliflozin Ameliorates Adverse Left Ventricular Remodeling in Nondiabetic Heart Failure by Enhancing Myocardial Energetics. J. Am. Coll. Cardiol..

[185] Von Lewinski D., Kolesnik E., Tripolt N.J., Pferschy P.N., Benedikt M., Wallner M., Alber H., Berger R., Lichtenauer M., Saely C.H. (2022). Empagliflozin in Acute Myocardial Infarction: The EMMY Trial. Eur. Heart J..

[186] Butler J., Jones W.S., Udell J.A., Anker S.D., Petrie M.C., Harrington J., Mattheus M., Zwiener I., Amir O., Bahit M.C. (2024). Empagliflozin after Acute Myocardial Infarction. N. Engl. J. Med..

[187] James S., Erlinge D., Storey R.F., McGuire D.K., De Belder M., Eriksson N., Andersen K., Austin D., Arefalk G., Carrick D. (2024). Dapagliflozin in Myocardial Infarction without Diabetes or Heart Failure. NEJM Evid..

[188] Bertolin-Boronat C., Marcos-Garcés V., Merenciano-González H., Perez N., Pérez Del Villar C., Gavara J., Lopez-Lereu M.P., Monmeneu J.V., Herrera Flores C., Domenech-Ximenos B. (2025). Prediction of Left Ventricular Thrombus after Myocardial Infarction: A Cardiac Magnetic Resonance-Based Prospective Registry. Eur. J. Intern. Med..

[189] Merenciano-González H., Marcos-Garcés V., Gavara J., Pedro-Tudela A., Lopez-Lereu M.P., Monmeneu J.V., Perez N., Rios-Navarro C., de Dios E., Gabaldón-Pérez A. (2022). Residual ST-Segment Elevation to Predict Long-Term Clinical and CMR-Derived Outcomes in STEMI. Sci. Rep..

[190] Marcos-Garcés V., Perez N., Gavara J., Lopez-Lereu M.P., Monmeneu J.V., Rios-Navarro C., de Dios E., Merenciano-González H., Gabaldon-Pérez A., Ferrero-De-Loma-Osorio Á. (2023). Cardiac Magnetic Resonance Outperforms Echocardiography to Predict Subsequent Implantable Cardioverter Defibrillator Therapies in ST-Segment Elevation Myocardial Infarction Patients. Front. Cardiovasc. Med..

[191] Pérez-Solé N., De Dios E., Gavara J., Ríos-Navarro C., Marcos-Garces V., Merenciano H., Climent J.I., López-Bueno L., Payá A., De La Espriella R. (2025). NT-proBNP to Guide Risk Stratification after Cardiac Rehabilitation in Patients with ST-Segment Elevation Myocardial Infarction. Eur. J. Intern. Med..

[192] Xu B., Li W., You Z., Yang N., Lin L., Li Y. (2024). Risk Factors for Left Ventricular Remodeling after Myocardial Infarction: A Meta-Analysis. Medicine.

[193] Gavara J., Rodriguez-Palomares J.F., Valente F., Monmeneu J.V., Lopez-Lereu M.P., Bonanad C., Ferreira-Gonzalez I., Garcia del Blanco B., Rodriguez-Garcia J., Mutuberria M. (2018). Prognostic Value of Strain by Tissue Tracking Cardiac Magnetic Resonance After ST-Segment Elevation Myocardial Infarction. JACC Cardiovasc. Imaging.

[194] Holmes A.A., Romero J., Levsky J.M., Haramati L.B., Phuong N., Rezai-Gharai L., Cohen S., Restrepo L., Ruiz-Guerrero L., Fisher J.D. (2017). Circumferential Strain Acquired by CMR Early after Acute Myocardial Infarction Adds Incremental Predictive Value to Late Gadolinium Enhancement Imaging to Predict Late Myocardial Remodeling and Subsequent Risk of Sudden Cardiac Death. J. Interv. Card. Electrophysiol..

[195] Reindl M., Tiller C., Holzknecht M., Lechner I., Eisner D., Riepl L., Pamminger M., Henninger B., Mayr A., Schwaiger J.P. (2021). Global Longitudinal Strain by Feature Tracking for Optimized Prediction of Adverse Remodeling after ST-Elevation Myocardial Infarction. Clin. Res. Cardiol..

[196] Yi L., Zhu T., Qu X., Buayiximu K., Feng S., Zhu Z., Ni J., Du R., Zhu J., Wang X. (2024). Predictive Value of Early Left Ventricular End-Diastolic Volume Changes for Late Left Ventricular Remodeling after ST-Elevation Myocardial Infarction. Cardiol. J..

[197] Bodi V., Monmeneu J.V., Ortiz-Perez J.T., Lopez-Lereu M.P., Bonanad C., Husser O., Minana G., Gomez C., Nunez J., Forteza M.J. (2016). Prediction of Reverse Remodeling at Cardiac MR Imaging Soon after First ST-Segment–Elevation Myocardial Infarction: Results of a Large Prospective Registry. Radiology.

[198] Troger F., Pamminger M., Poskaite P., Reindl M., Holzknecht M., Lechner I., Tiller C., Von Der Emde S., Kaser A., Oberhollenzer F. (2025). Clinical Impact of Persistent Microvascular Obstruction in CMR After Reperfused STEMI. Circ. Cardiovasc. Imaging.

[199] Bodi V., Sanchis J., Nunez J., Mainar L., Lopez-Lereu M.P., Monmeneu J.V., Rumiz E., Chaustre F., Trapero I., Husser O. (2009). Prognostic Value of a Comprehensive Cardiac Magnetic Resonance Assessment Soon after a First ST-Segment Elevation Myocardial Infarction. JACC Cardiovasc. Imaging.

[200] Berezin A.E., Berezin A.A. (2020). Adverse Cardiac Remodelling after Acute Myocardial Infarction: Old and New Biomarkers. Dis. Markers.

[201] Reinstadler S.J., Feistritzer H.-J., Klug G., Mair J., Tu A.M.-D., Kofler M., Henninger B., Franz W.-M., Metzler B. (2016). High-Sensitivity Troponin T for Prediction of Left Ventricular Function and Infarct Size One Year Following ST-Elevation Myocardial Infarction. Int. J. Cardiol..

[202] Weir R.A.P., Miller A.M., Murphy G.E.J., Clements S., Steedman T., Connell J.M.C., McInnes I.B., Dargie H.J., McMurray J.J.V. (2010). Serum Soluble ST2. A Potential Novel Mediator in Left Ventricular and Infarct Remodeling After Acute Myocardial Infarction. J. Am. Coll. Cardiol..

[203] Xu L., Huang X., Ma J., Huang J., Fan Y., Li H., Qiu J., Zhang H., Huang W. (2017). Value of Three-Dimensional Strain Parameters for Predicting Left Ventricular Remodeling after ST-Elevation Myocardial Infarction. Int. J. Cardiovasc. Imaging.

[204] Varasteh Z., Weber W.A., Rischpler C. (2022). Nuclear Molecular Imaging of Cardiac Remodeling after Myocardial Infarction. Pharmaceuticals.

[205] Gissler M.C., Antiochos P., Ge Y., Heydari B., Gräni C., Kwong R.Y. (2024). Cardiac Magnetic Resonance Evaluation of LV Remodeling Post-Myocardial Infarction. JACC Cardiovasc. Imaging.

[206] Holzknecht M., Reindl M., Tiller C., Lechner I., Perez Cabrera R., Mayr A., Brenner C., Klug G., Bauer A., Metzler B. (2021). Clinical Risk Score to Predict Early Left Ventricular Thrombus After ST-Segment Elevation Myocardial Infarction. JACC Cardiovasc. Imaging.

[207] Fonseca F.A.H., França C.N., Fonseca H.A.R., Serra A.J., Izar M.C. (2025). Key Inflammatory Players for Infarcted Mass and Cardiac Remodeling after Acute Myocardial Infarction. Front. Cardiovasc. Med..

[208] Matta A., Ohlmann P., Nader V., Moussallem N., Carrié D., Roncalli J. (2024). A Review of Therapeutic Approaches for Post-Infarction Left Ventricular Remodeling. Curr. Probl. Cardiol..

[209] Bauersachs J., Solomon S.D., Anker S.D., Antorrena-Miranda I., Batkai S., Viereck J., Rump S., Filippatos G., Granzer U., Ponikowski P. (2024). Efficacy and Safety of CDR132L in Patients with Reduced Left Ventricular Ejection Fraction after Myocardial Infarction: Rationale and Design of the HF-REVERT Trial. Eur. J. Heart Fail..

[210] Itzhar A., Yosef G., Eilon-Ashkenazy M., Shmidov Y., Gil H., Lacham-Hartman S., Elyagon S., Etzion S., Bitton R., Cohen S. (2023). Potent Inhibition of MMP-9 by a Novel Sustained-Release Platform Attenuates Left Ventricular Remodeling Following Myocardial Infarction. J. Control. Release.

